# A Computational Systems Biology Study for Understanding Salt Tolerance Mechanism in Rice

**DOI:** 10.1371/journal.pone.0064929

**Published:** 2013-06-07

**Authors:** Juexin Wang, Liang Chen, Yan Wang, Jingfen Zhang, Yanchun Liang, Dong Xu

**Affiliations:** 1 College of Computer Science and Technology, Jilin University, Changchun, China; 2 Digital Biology Laboratory, Computer Science Department, and Christopher S. Bond Life Sciences Center, University of Missouri, Columbia, Missouri, United States of America; University of Georgia, United States of America

## Abstract

Salinity is one of the most common abiotic stresses in agriculture production. Salt tolerance of rice (*Oryza sativa*) is an important trait controlled by various genes. The mechanism of rice salt tolerance, currently with limited understanding, is of great interest to molecular breeding in improving grain yield. In this study, a gene regulatory network of rice salt tolerance is constructed using a systems biology approach with a number of novel computational methods. We developed an improved volcano plot method in conjunction with a new machine-learning method for gene selection based on gene expression data and applied the method to choose genes related to salt tolerance in rice. The results were then assessed by quantitative trait loci (QTL), co-expression and regulatory binding motif analysis. The selected genes were constructed into a number of network modules based on predicted protein interactions including modules of phosphorylation activity, ubiquity activity, and several proteinase activities such as peroxidase, aspartic proteinase, glucosyltransferase, and flavonol synthase. All of these discovered modules are related to the salt tolerance mechanism of signal transduction, ion pump, abscisic acid mediation, reactive oxygen species scavenging and ion sequestration. We also predicted the three-dimensional structures of some crucial proteins related to the salt tolerance QTL for understanding the roles of these proteins in the network. Our computational study sheds some new light on the mechanism of salt tolerance and provides a systems biology pipeline for studying plant traits in general.

## Introduction

Salinity is one of agriculture’s most crucial problems in large parts of the world [1]. Rice (*Oryza sativa* L.), which provides a major food source for about half of the global population, is considered as the most important cereal crop in agriculture, but it is salt susceptible [2]. Soil salinity is a major abiotic stress, which limits rice production in about 30% of the rice-growing area worldwide [3], [4]. Under the heavy pressure of global population explosion and global climate change, studying rice salt tolerance is of high importance. Genetic improvements leading to salt tolerance of cereal crops in molecular breeding could help maintain stable global food supply [5]. Some traditional cultivars and landraces have been identified as tolerant to abiotic stresses, despite their undesirable agronomic traits such as tall plant stature, photosensitivity, poor grain quality and low yield. For example, Pokkali, an Indian landrace, can maintain high K+/Na+ ratio in shoot in a high salinity environment, and it could be a donor of salt-tolerance strains in breeding programs. FL478, an F2-derived F8, inherited the salt tolerance property in recombinant inbred lines from parents Pokkali and IR29. FL478 is also an improved indica cultivar used as a salt-susceptibility standard [6].

With years of continuous exploration, some general molecular mechanisms of salt tolerance in plants have been revealed. The high-salinity environment mainly disrupts the ironic and osmotic equilibrium of cells, and as a result, genes in several pathways are activated in response to high sodium concentration. Pathways related to ion pumps [7], calcium [8], SOS pathway [9], ABA (abscisic acid) [10], mitogen-activated protein kinases [11], glycine betaine [12], proline [13], reactive oxygen species [14], and DEAD-box helicases [15] are of significance in high salinity environment. They play different roles in maintaining high K+/Na+ ratio, synthesizing and segregating ions, and controlling ion concentration [16]. The genes and transcription factors that encode or regulate these components often demonstrate irregular activities in a high salinity environment. At the cell level, the most significant activities in dealing with excessive salt in plants is pumping ions out of a cell to keep the ion equilibrium, while the vacuole located in the cell helps store some ions. In salt-resistant detoxifying mechanisms, especially sequestration by vacuole [17], many salt tolerance genes with high level of activities in a high salinity environment are related to vesicle, membrane and ion transport. For example, H+-ATPase as a proton pump on cytoplasmic vesicle maintains the ion equilibrium of the cell by pumping H+ to the vacuole to retain pH and transmembrane proton gradient [18]; Na+ transporter plays an important role in maintaining high Na+/K+ ratio in various tissues [19], [20]. However, the global picture of salt tolerance mechanisms, especially rice-specific salt tolerance mechanisms is still unclear; for example, how ABA induces H_2_O_2_ control and how a plant transduces signals in response to salt tolerance are largely unknown.

Multiple sources of data can enhance the understanding of salt tolerance. The genetic variations of different rice responses to salt stress may shed some light on the roles of various genes in salt tolerance. The availability of rice genome sequencing [21], [22] further paved the way for in-depth study of rice salt tolerance. *Oryza sativa* microarray gene expression data have provided information on regulatory networks of salinity response. Kawasaki et al. analyzed the initial phase of salt stress in rice based on gene expression profiles [23]. Huang et al. identified a zinc finger protein named DST that regulates drought and salt tolerance in rice [24]. Zhang et al. studied OsGAPC3 over-expression in rice tolerance [25]. Mito et al. found that expression of DREB- and ZAT- related genes might be involved in the salt tolerance of the AtMYB102 chimeric repressor line [26]. Schmidit et al. examined transcription factors like heat shock factors (HSFs) in response to salinity environment and they characterized OsHsfC1b as playing a role in ABA-mediated salt stress tolerance in rice [27]. Nevertheless, these studies were mainly focused on a single gene or some isolated genes, and they lack systems-level understanding of the global molecular mechanism of salt tolerance given that salt resistance reacts in a coordinated and effective manner. In view of these findings, we conducted a systems-level study to fill the gap between isolated genes and the global mechanism of salt tolerance.

Among tens of thousands of genes in microarray data, it is challenging to choose the set of genes that are most relevant to salt tolerance [28], [29]. Biologists often use a volcano plot method, which reflects both fold of change and its statistical significance at the same time in a heuristic fashion [30]. However, such a method may not be sufficient to discover some complex relationships between genes and a certain phenotype, trait, or condition [31]. Some statistical methods for clustering and classification are extensively used to deal with this problem [32], [33]. Several machine-learning methods, such as random forest [34] and SVM-RFE (support vector machine recursive feature elimination) [35] were also developed for this purpose. RFE is commonly used for feature selection, and it eliminates features iteratively until getting the minimum subset of features. By combining SVM with the RFE procedure, SVM-RFE becomes an effective method in selecting and ranking genes in microarray data analyses [36], [37], [38]. In this study, we improved the volcano plot method using bootstrapping SVM-RFE to select informative genes related to salt tolerance from microarray datasets.

There are both challenges and advantages in analyzing rice data. For crops, there are typically limited experimental data available, and little bioinformatics work has been done on these data. On the other hand, crops, especially rice, have rich data related to traits, such as Quantitative Trait Locus (QTLs). QTLs are stretches of DNA containing or linked to genes that underlie a quantitative trait. It is a classical and widely used breeding method in identifying the actual genes underlying a trait in breeding experiments. With the availability of rice genome sequence, QTLs provide useful relationships between genes in a QTL region and its corresponding trait [39], [40]. In this work, we used QTLs in validating selected informative gene sets. We also used predicted protein-protein interactions to build a protein network of informative genes. Some crucial genes in the network with QTL evidence were studied by protein structure prediction, co-expression and gene regulatory motif analysis. Our computational study provides useful hypotheses for studying salt tolerance and may help improve molecular breeding of rice in salinity.

## Results

**Figure 1:**
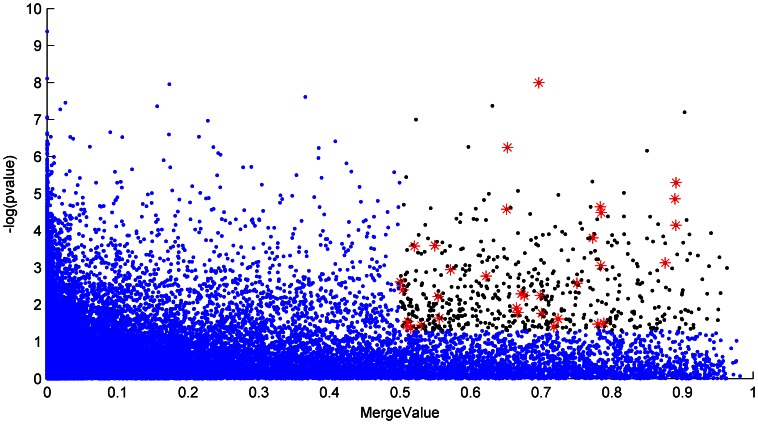
Improved volcano plot of GSE14403. The horizontal axis represents *MergeValue* obtained by bootstraps SVM-RFE. The vertical axis shows the –log(p-value) from t-test. Black dots indicate selected probes with *MergeValue* threshold of 0.5 and t-test p-value threshold of 0.05. Red stars indicate selected probes mapped on QTL region. Blue dots indicate unselected probes.

Using the microarray data, GSE14403, to compare salinity susceptible and resistant rice genotypes with the Gene Expression Omnibus (GEO), we chose the threshold as 0.05 in t-test p-value and 0.5 in *MergeValue* (as described in the Method section) to obtain 556 probes in the improved volcano plot in Figure 1. We assume that many of these 556 genes are related to salt tolerance, and they are listed in Table S1. Table 1 lists the gene enrichment result using AgriGO [41], a plant-specific GO term enrichment analysis. In molecular functions, the chosen genes are over represented in the categories of iron binding, cation binding, ion binding and heme binding–all of which may be active due to the high ion concentration in salinity. The significant behavior of these genes in oxidoreductase activity may be related to electron transport in complex chemical reactions that balances the charges during ion transport. The oxidoreductase activity may also be related to reactive oxygen intermediates (ROI) that are produced in response to oxidative stress due to a water deficit during salinity stress [14]. ROI can seriously disrupt normal metabolism through oxidative damage on lipids [42], [43], proteins and nucleic acids [42], [44]. The increased oxidoreductase activity is consistent with known activation of the antioxidative enzymes such as catalase (CAT), ascorbate peroxidase (APX), guaicol peroxidase (POD), glutathione reductase (GR), and superoxide dismutase (SOD) under salt stress in plants [16]. In biological processes, the cellular nitrogen compound metabolic process is over represented. As proline and glycine betaine accumulate under stress, they are correlated with osmotic adjustment to improve plant salinity tolerance [45]. Proline is also involved in scavenging free radicals, stabilizing subcellular structures and buffering cellular redox potential under stresses. Polyamines can be synthesized under salt tolerance [46]. In this sense, we speculate these nitrogen-containing compounds may be synthesized in “the cellular nitrogen compound metabolic process.” In cellular components, according to the gene enrichment analysis, most of the chosen genes are related to vesicle and membrane, which is consistent with the detoxifying mechanism of salt resistant genotypes, especially in sequestration by vacuole [17]. It is plausible to infer that some of these chosen proteins on membranes act as transporting ions outside the cell or to the vacuole to maintain pH, transmembrane proton gradient [18], and high K+/Na+ ratio [19].

**Table 1:** GO term enrichment analysis on gene selected from microarray by AgriGO.

GO term	Ontology	Description	Number inselected gene set	Number inBackground	p-value	FDR
GO: 0034641	P	cellular nitrogen compound metabolic process	17	459	2.40E-05	0.011
GO: 0005506	F	iron ion binding	19	432	7.10E-07	0.0002
GO: 0016491	F	oxidoreductase activity	32	1141	4.30E-06	0.0006
GO: 0043169	F	cation binding	51	2582	0.00014	0.01
GO: 0043167	F	ion binding	51	2584	0.00015	0.01
GO: 0020037	F	heme binding	9	205	0.00055	0.031
GO: 0046906	F	tetrapyrrole binding	9	217	0.00082	0.039
GO: 0031982	C	Vesicle	131	7454	3.40E-06	0.0001
GO: 0016023	C	cytoplasmic membrane-bounded vesicle	131	7445	3.20E-06	0.0001
GO: 0031988	C	membrane-bounded vesicle	131	7445	3.20E-06	0.0001
GO: 0031410	C	cytoplasmic vesicle	131	7454	3.40E-06	0.0001
GO: 0031224	C	intrinsic to membrane	23	820	8.10E-05	0.002
GO: 0016021	C	integral to membrane	21	804	0.0004	0.0083

Ontology “P” indicates Biological Process, Ontology “F” indicates Molecular Function, and Ontology “C” indicates Cellular Component. “Number in selected gene set” is the number of genes in the query gene list. “Number in background” is the number of genes in the proteome. P-value represents the statistical significance of the gene enrichment test. FDR means False Discovery Rate.

In order to assess the performance of improved volcano plot, we developed a Microarray-QTL test by using the QTL information as a criterion to evaluate the reliability of chosen genes. As shown in Table 2, our chosen genes are compared with the QTL regions mapped in the whole genome and these genes show high statistical significance in related QTL regions by our Microarray-QTL test. We also compared the performance of other feature selection methods in Document S1.

**Table 2:** Evaluation of choosing salt-tolerance genes by QTLs.

Methods	Sample Size	Original QTL region			Flanking region with length of the QTL region		
		Number of Hits	Percentage of Population	Microarray-QTLs testp-value	Number of Hits	Percentage of sample	Microarray-QTL testp-value
Improved Volcano Plot	556 selected	34	6.12%	4.21e-7	94	16.91%	7.44e-18

The Number of hits and the percentage of samples depict the number and percentage of the chosen genes that can be mapped into the (extended) QTLs regions. The Microarray-QTL test p-value means the statistical significance of these chosen genes according to the QTLs.

**Figure 2:**
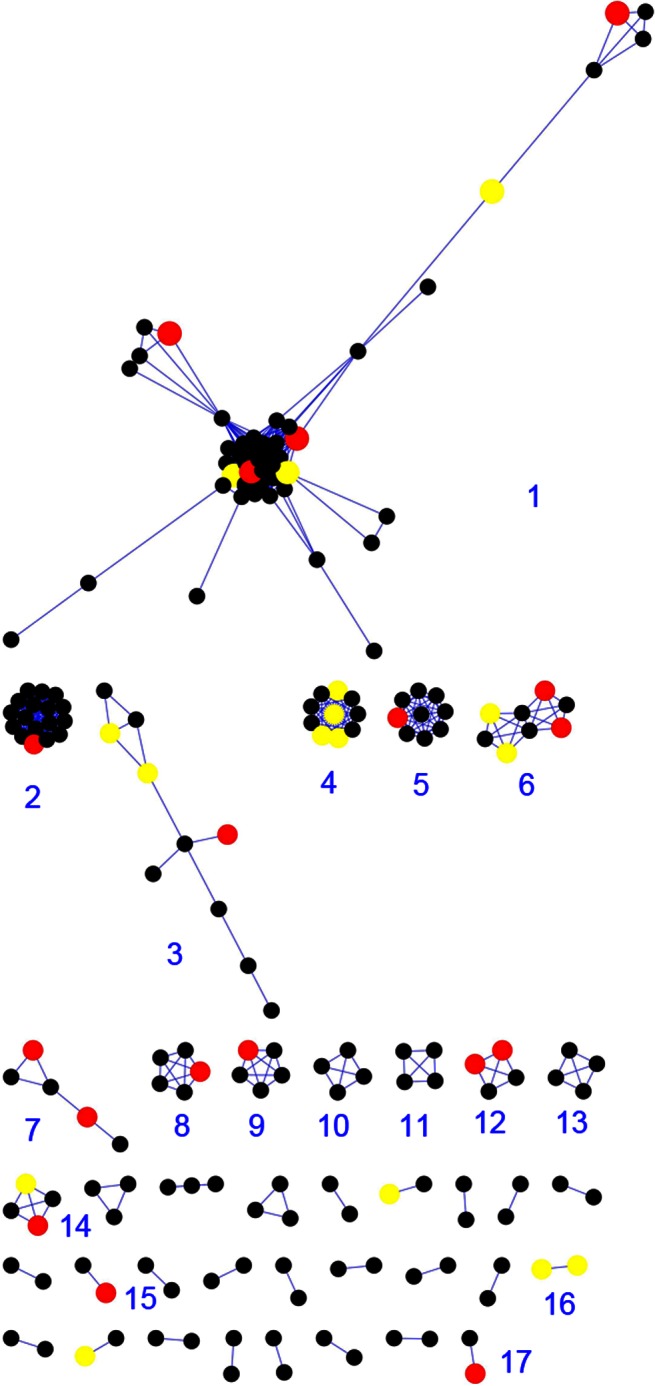
Salt-tolerance protein interaction modules that are related to QTLs. Red nodes represent the proteins located in QTL regions. Yellow nodes represent the proteins located in flanked regions with the lengths of the QTL regions.

We constructed a rice salt tolerance protein interaction network using the 556 genes selected by improved volcano plot methods as the nodes and protein-protein interaction data from DIPOS as the edges [47]. By merging the isoforms, the network contains 189 nodes and 705 edges, as visualized by Cytoscape [48] in Figure 2.

By analyzing the constructed network, we identified 17 modules with nodes located in QTL and flanked QTL region. Including flanked regions could help give some tolerance to the errors in QTL mapping [49]. Table 3 shows the functions of these identified modules related to known salt tolerance mechanisms.

**Table 3:** Modules of salt-tolerance network and their potential roles in salt tolerance.

NO	Function	Nodes	Edges	# of QTL hits	# of ExtendedQTL hits	Salt Tolerance Mechanism	QTL hits	Extended QTL hits
1	phosphorylation	47	372	4	7	Signal Transduction/ion homeostasis/ABA	LOC_Os01g61070.1, LOC_Os07g09420.1, LOC_Os07g12240.1, LOC_Os01g59580.1	LOC_Os01g70840.1/LOC_Os01g70840.2, LOC_Os09g29510.1, LOC_Os01g52640.3
2	cytochrome P450	17	136	1	1	ABA	LOC_Os09g26960.1	
3	NA	10	11	1	3	NA	LOC_Os07g08840.1	LOC_Os09g20220.1, LOC_Os01g70990.1
4	peroxidase	10	45	0	4	Antioxidate		LOC_Os06g33080.1, LOC_Os06g35480.1, LOC_Os06g33100.1, LOC_Os06g32990.1
5	aspartic proteinase nepenthesin	9	36	1	1	Agreement	LOC_Os01g64840.1	
6	Ubiquitin	8	19	2	4	ABA/degradation pathway	LOC_Os01g64620.1, LOC_Os01g60730.1/LOC_Os01g60730.2	LOC_Os09g31031.2, LOC_Os09g27930.1
7	starch	5	5	2	2	Agreement	LOC_Os04g33920.1, LOC_Os01g63810.1	
8	glucosyltransferase	5	10	1	1	Agreement	LOC_Os07g10190.1	
9	flavonol synthase	5	10	1	1	ROS-scavenging	LOC_Os01g61610.1/LOC_Os01g61610.2/LOC_Os01g61610.3	
10	glycosyl hydrolases family	4	6	0	0	Agreement		
11	MYB family transcription factor	4	6	0	0	Agreement		
12	gibberellin receptor GID1L2	4	6	2	2	Agreement	LOC_Os07g06840.1, LOC_Os07g06860.1	
13	O-methyltransferase	4	6	0	0	Sodium sequestration		
14	Phosphatidylethanolamine-binding	4	6	1	2	Cell signalling	LOC_Os04g33570.1, LOC_Os04g41130.1	
15	aldo/keto reductase family	2	1	1	1	Antioxidate	LOC_Os04g37490.1	
16	cytokinin dehydrogenase	2	1	0	2	ABA		LOC_Os01g56810.1, LOC_Os06g35650.1
17	cysteine synthase/indole-3-glycerol phosphate lyase	2	1	1	1	Agreement	LOC_Os06g36850.1	

The number of members and interactions of the module are shown in column of “Nodes and Edges.” “# of QTL hits” and “# of Extended QTL hits” depict the number of module members that can be mapped into the (extended) QTLs regions. “Salt tolerance mechanism” shows the process or essential component that could participate in salt tolerance response. “Agreement” means the component is only consistent with suggestions in other studies but there is no direct evidence to link it to known salt tolerance mechanism. “QTL hits” and “Extended QTL hits” illustrate the member of module mapped into the (extended) QTLs regions.

**Figure 3:**
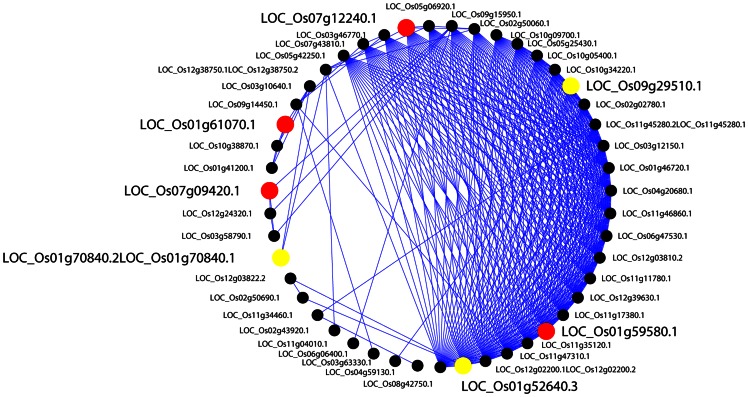
The largest module in the salt tolerance protein interaction network. Black nodes indicate genes covered by QTLs. Yellow nodes indicate genes covered by extended QTLs.

The largest module in this network contains 51 genes of 47 merged nodes and 372 edges. Figure 3 depicts the radical layout of this module and Table S2 shows the node annotation in detail. The GO enrichment analysis reveals related salt tolerance activities in Table 4, and protein phosphorylation represents the most significant function. It is known that protein phosphorylation plays a vital role in ion homeostasis under salinity stress in Arabidopsis [50], [51]. Under the salinity stress, phosphorylation often becomes active in signaling pathways; for example, MAPK transduces salt and other abiotic stress signals. In rice, Os-MAPK5 as a kinase can be triggered by salt, drought, wounding, cold, and ABA, resulting in an increase in tolerance to these abiotic stresses [10]. The Na+/H+ antiporter SOS1 mediates Na+ efflux. SOS2, a Ser/Thr protein kinase with N-terminal kinase catalytic domain regulates the activity of SOS1. SOS3, which senses salt stress-induced Ca2+ signature, thereby activating SOS2 to transduce the salt stress signal. Abscisic acid-mediated phosphorylation also plays a significant role in many activities within cytoplasmic proteins in rice under salinity stress [52]. The largest module that we identified includes these genes and others related to phosphorylation in nucleotide binding and kinase activities.

**Table 4:** AgriGO term enrichment analysis on the 51 genes in the largest module.

GO term	Ontology	Description	Number of genes	Number in Background	p-value	FDR
GO: 0016310	P	Phosphorylation	9	1080	4.9e-06	0.00033
GO: 0006468	P	protein amino acid phosphorylation	8	887	8.8e-06	0.00033
GO: 0006796	P	phosphate metabolic process	9	1206	1.2e-05	0.00033
GO: 0006793	P	phosphorus metabolic process	9	1206	1.2e-05	0.00033
GO: 0043687	P	post-translational protein modification	8	1236	8.8e-05	0.002
GO: 0034641	P	cellular nitrogen compound metabolic process	5	459	0.00016	0.0027
GO: 0006464	P	protein modification process	8	1359	0.00017	0.0027
GO: 0043412	P	macromolecule modification	8	1406	0.00021	0.003
GO: 0009069	P	serine family amino acid metabolic process	5	538	0.00034	0.0043
GO: 0044267	P	cellular protein metabolic process	8	2166	0.0031	0.033
GO: 0006520	P	cellular amino acid metabolic process	5	918	0.0034	0.033
GO: 0044106	P	cellular amine metabolic process	5	918	0.0034	0.033
GO: 0019538	P	protein metabolic process	9	2770	0.0041	0.036
GO: 0009308	P	amine metabolic process	5	1002	0.0049	0.041
GO: 0001883	F	purine nucleoside binding	9	1171	9.3e-06	0.00012
GO: 0001882	F	nucleoside binding	9	1171	9.3e-06	0.00012
GO: 0000166	F	nucleotide binding	11	1686	5.2e-06	0.00012
GO: 0005524	F	ATP binding	9	1071	4.6e-06	0.00012
GO: 0030554	F	adenyl nucleotide binding	9	1171	9.3e-06	0.00012
GO: 0032559	F	adenyl ribonucleotide binding	9	1074	4.7e-06	0.00012
GO: 0032555	F	purine ribonucleotide binding	9	1218	1.3e-05	0.00012
GO: 0032553	F	ribonucleotide binding	9	1218	1.3e-05	0.00012
GO: 0017076	F	purine nucleotide binding	9	1317	2.3e-05	0.00019
GO: 0016301	F	kinase activity	9	1464	5.1e-05	0.00039
GO: 0004672	F	protein kinase activity	7	1102	0.00026	0.0018
GO: 0016773	F	phosphotransferase activity, alcohol group as acceptor	7	1238	0.00051	0.0032
GO: 0004674	F	protein serine/threonine kinase activity	6	949	0.00069	0.004
GO: 0016772	F	transferase activity, transferring phosphorus-containing groups	9	2197	0.00091	0.005
GO: 0005488	F	Binding	21	8681	0.0015	0.0076

One gene can be in multiple GO families. In “Ontology”, “P” indicates biological process and “F” indicates molecular function.

**Figure 4:**
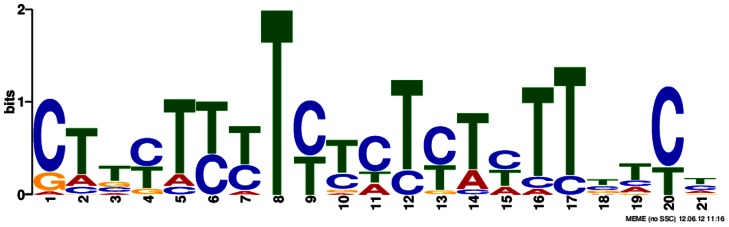
Detected motif in the upstream sequences of the largest module.

At the transcription level, we also checked whether the 51 genes in the largest module are transcriptionally co-regulated by examining if the promoter regions of these genes share conserved motifs as the regulatory elements. Three candidate motifs are predicted, and only one motif is validated by sequence comparison with known *cis* regulatory motifs in the PLACE database [53] as shown in Figure 4 and Table S3. This conserved motif is detected as “TCTCTCTCT”, the CTRMCAMV35S motif, which is a CT-rich motif found in a 60-nt region downstream of the transcription start site of the CaMV 35S RNA, and can enhance gene expression [54]. We also formed arbitrary reference gene sets selected randomly in whole genome scale and Chi-Square test on the validated motif showed significant statistical significance at p-value of 4.72e-13 (Table S4). We also checked the co-expression among the genes in this module. The averaged Pearson correlation coefficient of expression profiles inside the module is 0.402, comparing to 0.241 between genes in the module and randomly selected 51 genes in whole genome (Document S2). The motif analysis and co-expression analysis provide some support that this module is likely co-regulated.

**Figure 5:**
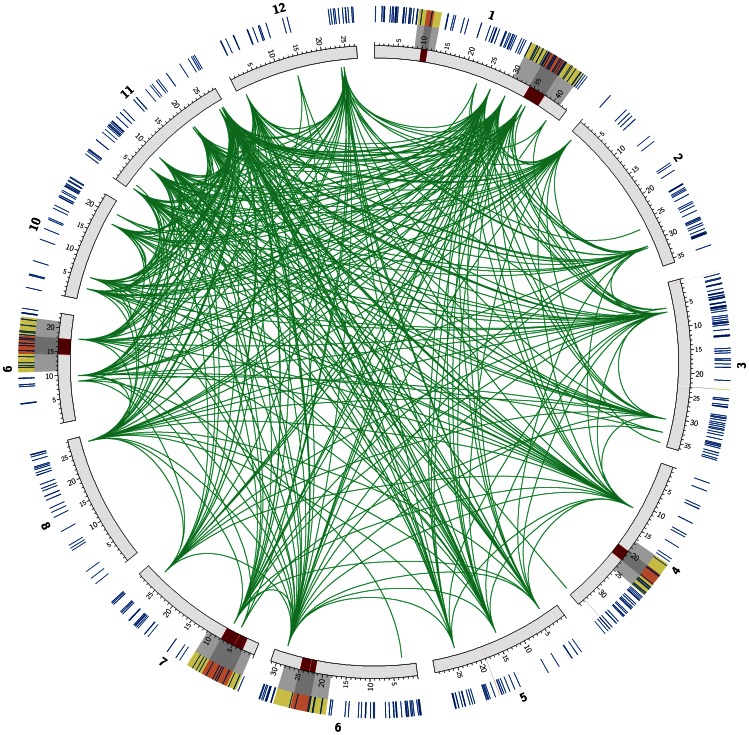
The whole genome mapping of selected genes and their interactions. Blue lines in the outer circle represent all the 556 selected genes. Red regions on the chromosome are the QTLs while the grey and yellow regions are the extended QTLs with one QTL length at each side of the flanking region. Inside the circle, green links show the protein-protein interactions among 51 genes in the largest module.

At the whole genome level, we mapped all 556 selected genes, QTL regions and extended QTL regions by their individual positions on the rice genome of MSU Rice Genome Annotation (Osa1) Release 6 [55] in Figure 5 using Circos [56]. Some selected genes are located in the QTL and extended QTL regions. We also mapped the 51 genes in the largest module on the genome, together with their interactions.

**Figure 6:**
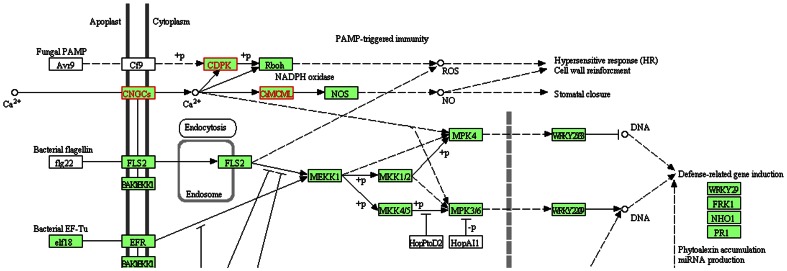
Part of the Arabidopsis Plant-Pathogen Interaction pathway in KEGG ( http://www.genome.jp/kegg-bin/show_pathway?ath04626+AT1G18210+AT3G51850+AT1G01340), where white boxes indicate that no genes have been assigned, green boxes have known genes in Arabidopsis, and boxes highlighted in red show the three mapped genes in the largest module.

We mapped the 51 genes of the largest module to the KEGG pathway (http://www.genome.jp/kegg/pathway.html). Three genes can be mapped to their Arabidopsis orthologs in the Plant-Pathogen Interaction Pathway [57], [58], as depicted in Figure 6. In this environmental adaptation pathway, CNGCs (Os02g0789100) is a cyclic nucleotide gated channel, CDPK (Os01g0622600) is a calcium-dependent protein kinase, and CaMCML (Os01g0505600) is the calcium-binding protein CML. All three of these proteins interact with each other and cooperate together in response to calcium ion signaling.

**Figure 7:**
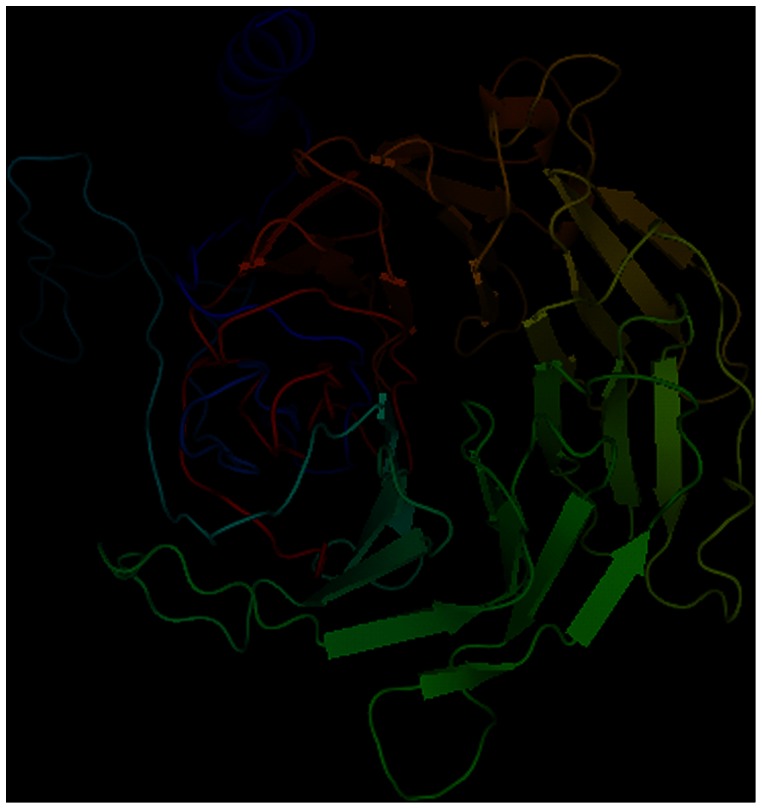
Predicted structural model of protein Os01g0725800.

One of the most interesting genes is LOC_Os01g52640.3, which is a hub gene in the largest module and overlaps with a QTL region. This gene corresponds to a hypothetical protein Os01g0725800, which interacts with 32 of the 51 proteins in the module. It contains four InterPro domains, namely, IPR000719, IPR001680, IPR011046, and IPR011009. IPR011009 domains can also be found in RIO kinase (IPR018935), a SPA1-related, serine/threonine-specific and tyrosine-specific protein kinase. This protein also has an ortholog in *Arabidopsis thaliana* as SPA4 (SPA1-RELATED 4), which is a binding protein and a signal transducer. We applied MUFOLD [59], [60] to predict the structure for LOC_Os01g52640.3. Using the identified templates of 2GNQ, 3EMH, and 3DM0 in PDB, we constructed the model for the protein region of 196–627 for the protein with the length of 432, as shown in Figure 7. The protein structural model contains the WD40 structure motif repeats, each with a typtophan-aspartic acid (W-D) dipeptide termination. As WD40 proteins often play important roles in signal transduction and transcription regulation [61], the structure prediction suggests that this protein may be related to signal transduction in the salt resistance process.

We also applied MUFOLD for structure predictions of hub proteins LOC_Os01g59580.1 and LOC_Os01g46720.1, each of which has a node degree of 30. According to the remote homology detection, LOC_Os01g59580.1 has a template 2QKW, which is in the process of phosphorylation (GO: 0006468), and plays a role as ATP binding (GO: 0005524), protein binding (GO: 0005515) and protein serine/threonine kinase activity (GO: 0004674) [62]. LOC_Os01g59580.1 may have these activities as well. LOC_Os01g46720.1 has templates 1PKD, 3EZR, 2W06 and 2W17, which are involved in anaphase-promoting complex-dependent, proteasomal ubiquitin-dependent protein catabolic process (GO: 0031145), and cyclin-dependent protein kinase activity (GO: 0004693). The details of predicted structural models are described in Document S3.

Besides exploring the largest module related to phosphorylation activity in a systems biology point of view, we also explored the other 16 identified modules in a similar fashion. Among these modules, Module 3 has divergent functions, but other modules converge to consistent functions related to salt tolerance. Module 2 of cytochrome P450 [63], Module 6 of ubiquitin activity [64], [65], and Module 16 of cytokinin dehydrogenase [66], [67] are related to ABA mediated in salt tolerance. Module 4 of peroxidase precursor [68], Module 9 of flavonol synthase [69], [70], and Module 15 of the aldo/keto reductase (AKR) family [71] are all related to ROS-scavenging in salt tolerance. Module 13 of O-methyltransferase [72] is associated with sodium sequestration. Some previous experimental studies on the abiotic stress of plants based on gene expression patterns also linked salt tolerance to aspartic proteinase nepenthesin [73] in Module 5, starch [74] in Module 7, glucosyltransferase [73] in Module 8, glycosyl hydrolases [75] in Module 10, MYB family [73] in Module 11, gibberellin receptor GID1L2 [76] in Module 12, phosphatidylethanolamine-binding [77] in Module 14, and cysteine synthase [78] in Module 17. A detailed analysis on each of these 16 modules can be found in Document S4.

## Discussion

In this paper, we focus on the salt tolerance mechanism of root tissue in rice. As there are commonly three samples in just one genotype of one condition on microarray experiments in contrast to tens of thousands of probe sets, it is a great challenge to determine feature selection on this small-sample, but high-dimension data. Classical statistical feature selection methods, such as t-test assume the samples follow some specific distribution as its hypothesis; however, the limited number of samples narrows the usage of these statistical methods. From the feature selection prospective, the volcano plot method uses two dimensions of fold change and t-test p-value to select genes in microarray analysis. It is a fast, simple, and widely used method. However, the fold change of each differential expression gene does not necessarily reveal the nature of biological meaning. In this case, using machine-learning methods could be a good alternative in microarray analysis. The improved volcano plot method using some specific criteria like *MergeValue* based on an SVM-RFE procedure could improve the performance. The improved method used all three salt resistant genotypes as a whole to mine the common pattern of salt tolerance, which helps overcome the disadvantage of a limited number of samples. The improved method also used a bootstraps approach to make the feature selection more robust.

We incorporated the QTL information with transcription profiles to identify genes related to drought response. For a given QTL, there may be 25–30 genes per cM (∼270 kbp in rice) [79]. Given so many possible genes in a QTL region associated to a phenotype, the proposed Microarray-QTL test, which used the same mechanism as GO term enrichment analysis, could help evaluate the relative relevance of these QTL genes to the phenotype quantitatively. Furthermore, we also combined predicted protein-protein interactions, protein structure prediction and gene regulatory motif analysis in studying potential genes related to salt tolerance. Such a systems approach is powerful in providing high-confidence predictions of salt-tolerant genes. Our study may provide richer and more concise predictions than a study done by Cotsaftis et al. [80], which only used the expression level of the gene probes in transcript profiling to predict salt-tolerant genes.

Walia et al. [5] summarized the following components in the salinity response based on their microarray study: 1) adaptive response, 2) non-adaptive response, 3) response to salt injury, and 4) heritable responses conferring tolerance. While these general categorizations are important, they do not provide a detailed mechanism, especially in terms of genes involved and these processes. Our study serves as an attempt to fill this gap. According to our constructed network, one kernel module of phosphorylation activity is detected. The role of phosphorylation in abiotic stress has been actively studied in recent years in addition to its relationship to salinity stress [81], cold stress [82] and heat stress [83]. Our result shows the central role of phosphorylation and redox action in salt tolerance, with implication of activity in signal transduction and oxidation. As our protein interaction networks were constructed from predicted interaction, our predictions of network modules may have significant false positives, which need further biological experiments to validate.

Our study may provide some useful hypotheses for researchers to design experiments for studying salt resistance and some guidance for molecular breeders to improve traits. Since some key proteins have been predicted and mapped to QTLs in our study, which means researchers could conduct experiments to clone and validate these genes. We plan to apply our computational pipeline to study other traits and other species.

## Materials and Methods

### Data Source

We obtained rice microarray data from Gene Expression Omnibus (GEO, http://www.ncbi.nlm.nih.gov/geo/), which contains 14 datasets, 182 platforms, 5,210 samples and 374 series of *Oryza sativa*. Among these datasets, we used GSE14403 submitted by Ute Baumann January 13, 2009 and last updated August 2, 2012 to analyze salt tolerance. Unlike other datasets such as GSE3053 and GSE13735, this dataset contains the largest size of samples ever gathered related to salt tolerance of roots. The data were generated from Affymetrix Rice Genome Array (GPL 2025 in GEO), which contains 57,381 probes and each probe corresponded to an individual gene.

In the dataset GSE14403, we used salt resistant genotypes FL478, Pokkali and IR63731, and salt susceptible genotype IR29 under control and salinity-stressed conditions during vegetative growth, which ranged from GSM359902 to GSM359924. We merged these three salt-tolerant plants together as the salt-tolerant group. In the microarray experiments that collected the data [80], seedlings were cultured in sand and irrigated with a nutrient solution for 22 days (salt-treated) and 30 days (control) after germination, respectively. Salinity treatment was applied by adding NaCl and CaCl_2_ (5∶1 molar concentration) in two steps over a period of 3 days (final electrical conductivity: 7.4 dS m−1) to prevent osmotic shock. All plants were harvested on day 30. All the data were collected from the root tissue of the plants. Table 5 gives the specific description of the data used, and the class label (−1/1) is according to the different stress conditions. Table 6 shows 17 QTLs (13 unique QTLs) detected with salt tolerance in Gramene (http://www.gramene.org/qtl/). We mapped these QTLs regions using Gramene annotation of the rice genome of MSU Rice Genome Annotation (Osa1) Release 6 [55].

**Table 5:** GSE14403 resistant samples description.

No	Sample	Genotype	Repeats	Stress condition	Class Label
1	GSM359902	FL478	1	Control	+1
2	GSM359903	FL478	2	Control	+1
3	GSM359904	FL478	3	Control	+1
4	GSM359905	FL478	1	Salt-treated	−1
5	GSM359906	FL478	2	Salt-treated	−1
6	GSM359907	FL478	3	Salt-treated	−1
7	GSM359913	IR63731	1	Control	+1
8	GSM359914	IR63731	2	Control	+1
9	GSM359915	IR63731	3	Control	+1
10	GSM359916	IR63731	1	Salt-treated	−1
11	GSM359917	IR63731	2	Salt-treated	−1
12	GSM359918	IR63731	3	Salt-treated	−1
13	GSM359919	Pokkali	1	Control	+1
14	GSM359920	Pokkali	2	Control	+1
15	GSM359921	Pokkali	3	Control	+1
16	GSM359922	Pokkali	1	Salt-treated	−1
17	GSM359923	Pokkali	2	Salt-treated	−1
18	GSM359924	Pokkali	3	Salt-treated	−1

**Table 6:** QTLs related to salt tolerance in rice from the Gramene QTL library.

No.	QTL name	Chr	Mapped positions	Note
1	AQEM001	1	33,956,950–37,713,775	
2	AQEM006	1	9,820,009–11,232,822	
3	AQEM008	1	33,956,950–37,713,775	Same as 1
4	AQGR001	1	38,530,957–38,531,467	
5	AQGR002	3	22,798,284–22,830,744	
6	AQCL001	3	484,860–485,333	
7	AQCL002	4	33,663,984–33,664,487	
8	AQEM009	4	19,928,370–22,355,854	
9	AQCL003	5	18,874,932–18,875,558	
10	AQEM002	6	21,605,889–24,919,236	
11	AQCL004	6	22,862,400–22,862,821	
12	AQEM003	7	4,573,316–7,739,951	
13	AQEM004	7	2,633,784–4,575,215	
14	AQEM010	7	2,633,784–4,575,215	Same as 13
15	AQEM005	7	2,633,784–4,575,215	Same as 13
16	AQEM011	7	2,633,784–4,575,215	Same as 13
17	AQEM007	9	14,362,062–17,837,010	

We obtained predicted protein-protein interaction data from Database of Interacting Proteins in *Oryza Sativa* (DIPOS) (http://csb.shu.edu.cn/dipos/) [47]. This database used two different but complementary methods, i.e., interologs and domain interactions based methods to predict protein interactions for rice. DIPOS contains 14,614,067 pairwise interactions among 27,746 proteins, covering about 41% of the whole *Oryaza sativa* proteome.

### Data Preprocessing

The preprocessing step intended to overcome the noises, including missing probes and mislabeled probes. We conducted the analysis using Bioconductor [84]. We used Bioconductor’s affy package for estimation of expression values by Robust Multi-chip Average (RMA) [85]. The RMA procedure consists of three steps: a background adjustment, quantile normalization and summarization. As there are 123 probe sets designed for control in this microarray, the dataset excluded these probe sets and 57,258 valid probe sets were obtained for further analysis.

### Choice of Gene List using Improved Volcano Plot

We proposed an improved volcano plot method to choose genes in this dataset. The improved method has a new measure *MergeValue* for selecting genes. The detail of this method is described in Document S5.

### Validate Gene List from QTL

Firstly, we identified QTLs zone in the genome using Gramene QTL database (http://www.gramene.org/qtl/), where there are 17 QTLs ranging from chromosomes 1,3,4,5,6,7,9. Secondly, we mapped the candidate genes detected by the microarray probe sets to the genome. Thirdly, we defined a criterion named Microarry-QTLs (Eq.(1)) to evaluate the statistical significance of chosen genes related with the specific trait.
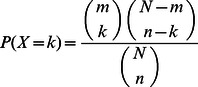
(1)where *N* is the total number of valid genes in the microarray, *m* is number of valid genes covered by QTLs, *n* is the number of chosen genes by microarray feature selection method, and *k* is the number of chosen genes that are covered by QTLs. The Microarray-QTL test follows a Hypergeometric distribution, and the p-value reveals significance of chosen genes related with specific QTLs.

### Motif Analysis on Promoters

To determine whether chosen genes are transcriptionally co-regulated, a motif analysis could help to find conserved motif regulatory elements in their promoters. We first used MEME [86] to predict motifs on the upstream region of 1,000 bps from the translation start site of the chosen genes. Then we used FIMO [87] to conduct a Chi-squared test on the significance of these motifs in comparison to randomly selected genes in the genome. We compared the identified motifs with known plant motifs from the PLACE database [53] using CompariMotif [88]. For each pair of compared motifs, if their similarity score is more than 4 and the percentage of their matched positions is more than 80%, these two motifs were considered identical.

## Supporting Information

Table S1 **556 genes selected by Improved Volcano Plot.**
(XLS)

Table S2 **51 genes in the largest module.**
(XLS)

Table S3 **Motif of the largest module discovered by PLACE database.**
(XLS)

Table S4 **Chi-Square test on the validated motif of the largest module.**
(XLS)

Document S1 **Feature selection comparison of improved volcano Plot.**
(DOC)

Document S2 **Supplementary of Co-expression analysis.**
(DOC)

Document S3 **Supplementary of Protein Prediction by MUFOLD.**
(DOC)

Document S4 **Annotation for modules identified from the constructed network.**
(DOC)

Document S5 **Choice of gene list using improved volcano plot.**
(DOC)
