# Supplementary material for: A Computational Systems Biology Study for Understanding Salt Tolerance Mechanism in Rice
Source: PLoS One. 2013 Jun 7;8(6):e64929. doi: 10.1371/journal.pone.0064929 (PMC3676415; doi:10.1371/journal.pone.0064929)
Supplement: Document S1 — Feature selection comparison of improved volcano Plot. (DOC) [file pone.0064929.s005.doc]

**Document S1:**

**Feature selection comparison of improved volcano Plot**

In order to assess the performance of improved volcano plot, we developed a Microarray-QTL test by using the QTL information as a criterion to evaluate the reliability of chosen genes. A standard volcano plot method was implemented by setting fold change >2 and t-test p-value <0.05, and it chose 244 probes. In comparison with single feature selection methods for microarray data such as t-test and the proposed *MergeValue* based on the bootstrap SVM-RFE feature selection, the proposed improved volcano plot method shows much better performance in the Microarray-QTL test. As shown in Table A, the different sets of chosen genes by different feature selection methods are compared with the QTL regions mapped in the whole genome. The assessment of whether the feature selection method chose appropriate genes is defined by the criterion of the number of hits of chosen genes, percentage of population and the statistical significance of Microarray-QTL test. In all three criteria, the improved volcano plot obtained the best results, which means the chosen genes sets by this method have the most convincing meaning evaluated by salt-tolerance QTLs.

| Methods | Sample Size | Flanking region with length of one gene | | | Flanking region with length of the QTL region | | |
| --- | --- | --- | --- | --- | --- | --- | --- |
|  |  | Number of Hits | Percentage of Population | Microarray-  QTLs test p-value | Number of Hits | Percentage of sample | Microarray-QTL test p-value |
| t-test | Top 500 | 28 | 5.60% | 1.85e-5 | 68 | 13.60% | 3.27e-9 |
| Volcano Plot | 244 selected | 14 | 5.74% | 1.40e-3 | 40 | 16.39% | 3.53e-8 |
| Improved Volcano Plot | 556 selected | **36** | 6.47% | 5.15e-8 | **91** | 16.37% | 1.93e-16 |

Table A. Evaluation of various methods for choosing salt-tolerance genes by QTLs. In the “Methods” column, t-test, *MergeValue*, volcano plot and improved volcano plot are used to choose feature genes. According to different settings, these four methods chose different numbers of genes as the sample sizes. The number of hits and the percentage of sample depict the number and percentage of the chosen genes that can be mapped into the (extended) QTLs regions. The Microarray-QTL test p-value means the statistical significance of those chosen genes according to the QTLs. Bold font highlights provide the best result.
