# Supplementary material for: A Computational Systems Biology Study for Understanding Salt Tolerance Mechanism in Rice
Source: PLoS One. 2013 Jun 7;8(6):e64929. doi: 10.1371/journal.pone.0064929 (PMC3676415; doi:10.1371/journal.pone.0064929)

**Document S2:**

**Supplementary of Co-expression analysis**

In order to validate the 51-genes module, we randomly chose 51 genes from genes outside the module as the reference set. In the heatmap below, comparing with the tailed reference set, we could clearly see the left-corner box of 51 genes within the module has a clear pattern standing out from the background.


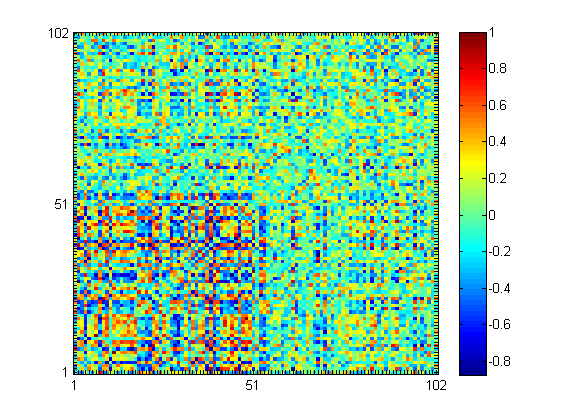

Supplement: Document S2 — Supplementary of Co-expression analysis. (DOC) [file pone.0064929.s006.doc]
