# Supplementary material for: A Computational Systems Biology Study for Understanding Salt Tolerance Mechanism in Rice
Source: PLoS One. 2013 Jun 7;8(6):e64929. doi: 10.1371/journal.pone.0064929 (PMC3676415; doi:10.1371/journal.pone.0064929)
Supplement: Document S3 — Supplementary of Protein Prediction by MUFOLD. (DOC) [file pone.0064929.s007.doc]

**Document S3:**

**Supplementary of Protein Prediction by MUFOLD**

We use MUFOLD [1][2] to predict the protein structure of the crucial proteins in the biggest module of the network. We were mainly concerned the hubs in the module, which has the most degrees. Besides Os01g0725800 explored in the main article, we also performed protein structure prediction on four other proteins of preferred interest.

LOC_Os01g59580.1 (probe OsAffx.985.1.S1_x_at) is expressed from the gene Os01g0810900, and it has degree of 30. As annotated by Affymetrix, it is a protein kinase-like domain containing protein, and it contains InterPro domains: IPR000719, IPR001245, IPR002290, IPR008271, and IPR011009, which recognize and bind Glc2-N-glycan, serine/threonine-specific and tyrosine-specific protein kinases based on its annotation. MUFOLD remote homolog search found 2QKW in PDB, which is an avirulence protein known as protein kinase (E.C.2.7.11.1). According to the Gene Ontology annotation, this protein involves in the process of phosphorylation (GO:0006468 ), and plays a role as ATP binding (GO:0005524), protein binding (GO:0005515 ) and protein serine/threonine kinase activity (GO:0004674 ). The predicted model by MUFOLD is shown in Figure A. This protein likely plays a role in the process of phosphorylation.


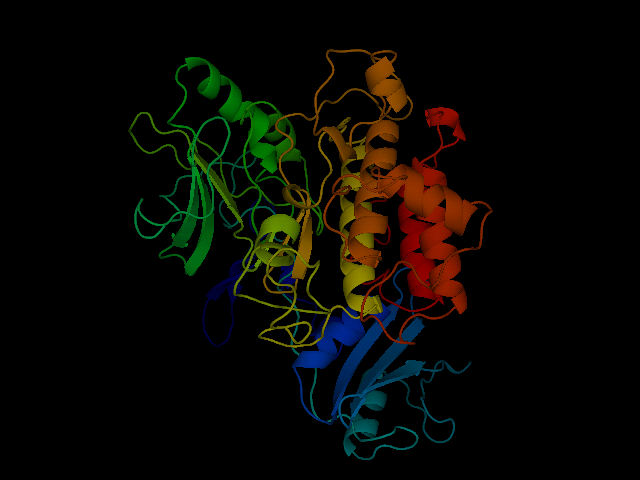


Figure A. Predicted structure model of protein LOC_Os01g59580.1

The InterPro domains of LOC_Os01g46720.1 (probe Os.27759.1.S1_at) known asIPR000719 and IPR011009 are annotated as plastid lipid-associated proteins (PAPs), Serine/Threonine protein kinases, and RIO kinases. LOC_Os01g46720.1 also has ortholog in *Arabidopsis thaliana* as ORG1 (OBP3-responsive gene 1) as ATP binding / kinase/ protein kinase. By applying predicted secondary structure and solvent accessibility, proteins 1PKD, 3EZR, 2W06 and 2W17 as cell division protein kinase 2 examples could serve as templates. The structural model predicted by MUFOLD is shown in Figure B. The protein 1PKD is detected as the main template by MUFOLD, and it is cell division protein kinase 2 (E.C.2.7.1.-), Cyclin A2, and EC cyclin-dependent kinase or protein-serine/threonine kinase (2.7.11.22 ). Annotated by Gene Ontology, this homology is involved in a biological process anaphase-promoting complex-dependent proteasomal ubiquitin-dependent protein catabolic process (GO:0031145), and functions in DNA damage response, acting as a signal transduction by p53 class mediator resulting in cell cycle arrest (GO:0006977) and DNA replication (GO:0006260), and function as molecular function ATP binding (GO:0005524 ), cyclin-dependent protein kinase activity ( GO:0004693 ) and identical protein binding (GO:0042802 ). According to MUFOLD, the structure information of LOC_Os01g46720.1 is also consistent with our hypothesis.


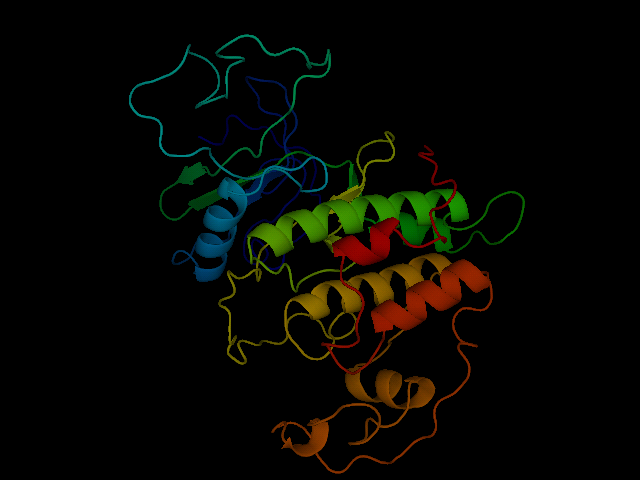


Figure B. Predicted structure model of protein LOC_Os01g46720.1

LOC_Os12g39630.1 (probe Os.15247.1.S1_s_at) encoded by Os12g0586100 is an expressed CAMK including calcium/calmodulin dependent protein kinases, which also has degree of 30 in this module. The GO annotation reveals its activities in response to osmotic stress and abscisic acid mediated signaling pathway. LOC_Os12g39630.1 also has ortholog in *Arabidopsis thaliana*, which is OST1 (OPEN STOMATA 1) as calcium-dependent protein serine/threonine kinase/ kinase/ protein kinase. MUFOLD detected 3HZT in PDB as a highly confident homology and built the model based on the sequence-structure alignment between LOC_Os12g39630.1 and 3HZT in Figure C. 3HZT is a calcium-dependent protein kinase 3 (E.C.2.7.11.17), and it functions as an EC Ca2+/calmodulin-dependent protein kinase; it also plays a role in transferases and in transferring phosphorous-containing groups. It is protein-serine/threonine kinases (2.7.11.17).


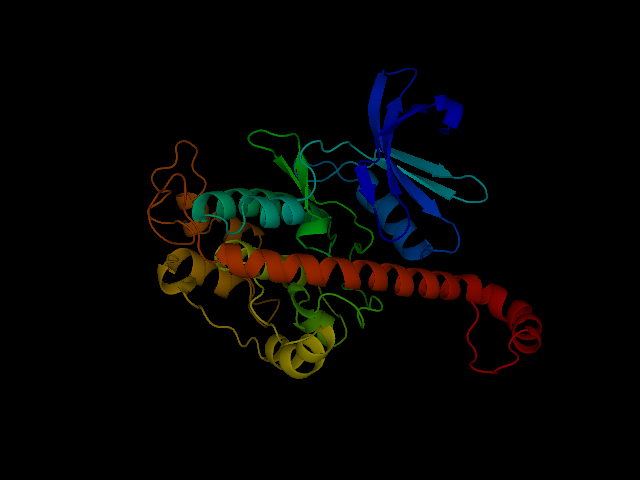


Figure C. Predicted structure model of protein LOC_Os12g39630.1

LOC_Os12g03810.2 (probe Os.10274.4.S1_at) encoded by Os12g0132200 fails in mapping QTL zone but its degree is still 30. It is also an expressed CAMK including calcium/calmodulin dependent protein kinases. The GO annotation reveals that it acts in the process of signal transduction. LOC_Os12g03810.2 also has ortholog CIPK3 (CBL-INTERACTING PROTEIN KINASE 3) in *Arabidopsis thaliana* as kinase/ protein kinase/ protein serine/threonine kinase. MUFOLD also detected 3HZT as the homology of LOC_Os12g03810.2 and built the structural model, which is shown in Figure D, which may act similar or be compared to Os12g0586100 in transferring phosphorous-containing groups.


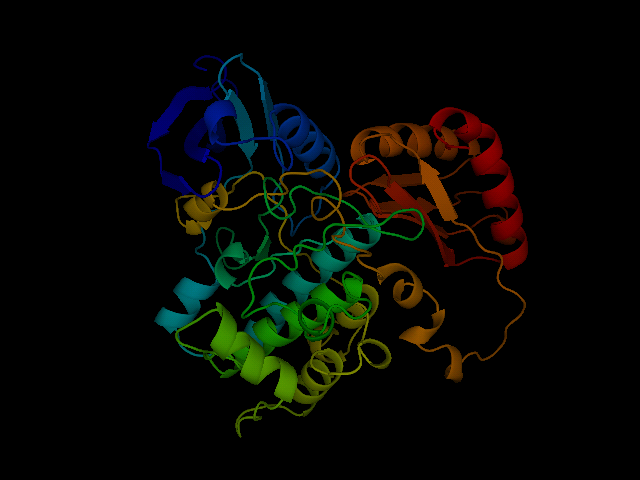


Figure D. Predicted structure model of protein LOC_Os12g03810.2

**References:**

[1] Zhang J, Wang Q, Barz B, He Z, Kosztin I, Shang Y, Xu D (2010) MUFOLD: A new solution for protein 3D structure prediction. Proteins 78(5):1137-1152.

[2] Zhang J, Wang Q, Vantasin K, Zhang J, He Z, Kosztin I, Shang Y, Xu D (2011) A multi-layer evaluation approach for protein structure prediction and model quality assessment. Proteins; Volume 79, Issue Supplement S10: 172–184.
