# Supplementary material for: A Computational Systems Biology Study for Understanding Salt Tolerance Mechanism in Rice
Source: PLoS One. 2013 Jun 7;8(6):e64929. doi: 10.1371/journal.pone.0064929 (PMC3676415; doi:10.1371/journal.pone.0064929)
Supplement: Document S4 — Annotation for modules identified from the constructed network. (DOC) [file pone.0064929.s008.doc]

**Document S4**

**Annotation for modules identified from the constructed network.**

In this document, we will give a detailed description of the 17 modules identified from the constructed network. In the main text, we have already used GO enrichment analysis, QTL statistical analysis, promoter motif analysis, pathway analysis and protein structure analysis on the largest module (i.e., Module 1), which has 51 genes. However, for the other 16 modules, the number of genes in a module is small, which makes it impossible to conduct the same extensive analysis. Hence, we mainly used gene annotation of Rice Genome Annotation Project MSU7 [1] to explore these modules. Table A shows all the annotations of members of these 16 modules. Table B shows co-expression analysis of these modules. Figure A shows the global interactions of these modules on the genome.

Module 2 of the constructed network has 17 members and 136 interactions. These genes have significant p-values in biological process of electron transport (GO:0006118), and molecular function monooxygenase activity (GO:0004497) based on the gene enrichment analysis (Table C). All these nodes are related to the cytochrome P450 (CYP) superfamily, which is a group of enzymes acting as monooxygenases that catalyze the oxidation of organic substances [2]. Under the salinity stress of plants, CYP is important in the biosynthesis of hormones and signaling molecules as well as in xenobiotic metabolism and detoxification [3]. CYP inhibitors performs significant chemical regulation on abscisic acid (ABA) catabolism in plants [4], where ABA plays an important role in salinity stress. CYP demonstrates significant changes in the transcription level under stresses by monitoring the expression of Arabidopsis. Seki et al. [5] and Narusaka et al. [6] both showed CYP were down-regulated under stresses.

Module 3 is very sparse with only 11 interactions for 10 nodes. As the annotations of genes of that module diverge, inference from gene enrichment analysis is not meaningful.

Module 4 which has 10 member and 45 interactions is related to peroxidase precursor. These genes have significant p-values in biological process of response to oxidative stress(GO:0006979), and molecular functions peroxidase activity (GO:0004601) and antioxidant activity (GO:0016209) (Table D). Under the salinity stress, peroxidase decomposes H2O2 by oxidation of co-substrates such as phenolic compounds and/or antioxidants [7]. As early as 1984, Kalir et al. researched the relationship between peroxidase and catalase activity in leaves of halimione portulacoides exposed to salinity [8]. Not only does peroxidase function in the metabolism of reactive oxygen, it is also involved in biosynthesis of the cell wall [9] including lignification and suberization [10, 11].

Module 5 is related to aspartic proteinase nepenthesin, which has 9 members and 36 interactions. Aspartic proteases are a family of protease enzymes that use an aspartate residue for catalysis of their peptide substrates [12], and it may have a role in the defense response of potatoes [13]. Aspartic proteinase also showed that it could be significantly down-regulated under salt stress in Arabidopsis [5].

Module 6 is related to ubiquitin with 8 members and 19 interactions. Four of the eight proteins in this module are located in the QTL region. Ubiquitin is a small regulatory protein directing proteins to compartments in the cell, including the proteasome that destroys and recycles proteins [14]. Ubiquitin proteins such as OsRDCP1 in rice cells could enhance the cellular level of stress-responsive proteins by accelerating the ubiquitin-dependent degradation of certain transcriptional repressor(s), and it also induces subsequent degradation pathways of proteins responsible for the inactivation or degradation of water stress-related proteins, which would also result in increased levels of defense proteins in rice cells [15, 16]. A number of ubiquitin-related genes have been validated to enhance salt tolerance, such as XERICO RING E3 ligase. Under high salt and osmotic stress, transcription-level increase of this gene could increase ABA levels [17, 18]. It also demonstrated significant expression in ubiquitin proteins in Arabidopsis under salinity stress [5].

Module 7 is also a sparse module with 5 members and 5 interactions, and it is related to starch. Starch accumulation was noticed in salt-tolerant rice exposed to the salinity stress [19]. This mechanism may result from the increased activity of alkaline invertase, which hydrolyzes sucrose and converts it into simpler sugars. Starch may be synthesized from such sugars. The ability of plants to partition sugars into starch may help avoid metabolic alterations by lowering feedback inhibition caused by excess amount of sucrose in cytoplasm [20, 21]. This accumulation of starch is also reported in salt-tolerant tomato cultivars [22].

Module 8 is related to glucosyltransferase with 5 members and 10 interactions. OsGGT (Oryza sativa glycogenin glucosyltransferase) could be related to submergence stress and associated with a general defensive response to various environmental stresses [23, 24]. Glucosyltransferase also becomes up-regulated in Arabidopsis under the salinity stresses [5].

Module 9 is related to flavonol synthase with 5 members and 10 interactions. Flavonoids constituting a secondary ROS-scavenging system in plants are exposed to severe/prolonged stress conditions. As H2O2 generated under the salinity stress is transported to the vacuole, the vacuole in the ROS homeostasis might be mediated by flavonoids [25-27].

Module 10 is in the glycosyl hydrolases family. Zhou et al. also demonstrated significant differential expression of rice in response to drought and high-salinity stresses [28].

Module 11 is related to MYB family transcription factor, which also becomes up-regulated in Arabidopsis when exposed to salinity stress [5].

Module 12 is related to gibberellin receptor GID1L2. In a transgenic Arabidopsis, overexpression of the salinity-responsive dwarf and delayed flowering 1 (DDF1) gene, encoding an AP2 transcription factor of the DREB1/CBF subfamily causes dwarfism mainly by reducing expression of bioactive gibberellin [29]. Ogawa et al.’s study on rice under the cadmium stress also found significant change in expression of gibberellin [30].

Module 13 is related to O-methyltransferase, which is involved in suberin biosynthesis [31], while suberin is a component of the polymer matrices in lipophilic cell wall barriers. These barriers control the fluxes of gases, water, and solutes, and they also help to protect plants from biotic and abiotic stresses and to control plant morphology [32]. Sheveleva et al. used transgenic tobacco plants carrying a cDNA encoding myo-inositol O-methyltransferase (IMT1), which showed increased recovery under drought and salt stress [33]. O-methyltransferase is also differentially expressed by microarray analysis of salinity-stressed barley plants [34].

Module 14 is associated with phosphatidylethanolamine-binding. Phosphatidylethanolamine-binding proteins in other organisms have been reported to be involved in the regulation of cell signaling [35].

Module 15 is related to the aldo/keto reductase (AKR) family. AKR family proteins often act as oxidoreductase dealing with reactive oxygen species in salt tolerance mechanism of rice. One possible procedure of improving tolerance against oxidative and heat stress is by overproduction of a rice aldo–keto reductase using malondialdehyde and methylglyoxal detoxification [36]. It was also found that expression of AKR4C9 in AKR family was markedly upregulated by osmotic stress, salinity, oxidative stress, and infection [37].

Module 16 is related to cytokinin dehydrogenase. Changes in ABA status can regulate shoot cytokinin concentrations via altering their metabolism [38]. High-throughput gene expression studies revealed that under exogenous ABA treatments, the key cytokinin biosynthesis gene IPT was down-regulated and cytokinin degradation CKX gene was upregulated in guard cells [39, 40].

Module 17 is related to cysteine synthase and indole-3-glycerol phosphate lyase. Cysteine synthase is differentially expressed in the salinity tolerance of wheat [41] and is a key player during Al response/adaptation in rice [42]. Indole-3-glycerol phosphate lyase in amino acid metabolism is up-regulated in response to abiotic stress [43, 44].

Table A. Gene members of Modules 2-17 in salt-tolerance network.

| Module | Gene | MSU7 Annotation | QTL |
| --- | --- | --- | --- |
| 2  Cytochrome P450 | LOC_Os10g30410.1 | cytochrome P450 71D7, putative, expressed |  |
|  | LOC_Os02g30100.1 | cytochrome P450, putative, expressed |  |
|  | LOC_Os08g43390.1 | cytochrome P450, putative, expressed |  |
|  | LOC_Os11g04290.1 | cytochrome P450, putative, expressed |  |
|  | LOC_Os02g44654.1LOC_Os02g44654.2 | cytochrome P450, putative, expressed |  |
|  | LOC_Os02g32770.1 | cytochrome P450, putative, expressed |  |
|  | LOC_Os02g12680.1 | cytochrome P450, putative, expressed |  |
|  | LOC_Os01g43720.1 | cytochrome P450 72A1, putative, expressed |  |
|  | LOC_Os01g08810.1 | cytochrome P450, putative, expressed |  |
|  | LOC_Os06g19070.1 | cytochrome P450, putative, expressed |  |
|  | LOC_Os10g05020.1 | cytochrome P450, putative, expressed |  |
|  | LOC_Os01g43774.1 | cytochrome P450 72A1, putative, expressed |  |
|  | LOC_Os06g02019.1 | cytochrome P450, putative, expressed |  |
|  | LOC_Os08g39694.1LOC_Os08g39694.2 | cytochrome P450, putative, expressed |  |
|  | LOC_Os03g55240.1 | cytochrome P450, putative, expressed |  |
|  | LOC_Os10g38110.1 | cytochrome P450, putative, expressed |  |
|  | LOC_Os09g26960.1 | cytochrome P450, putative, expressed | Q |
| 3  NA | LOC_Os10g38600.1 | glutathione S-transferase GSTU6, putative, expressed |  |
|  | LOC_Os09g20220.1 | glutathione S-transferase, putative, expressed | Qex |
|  | LOC_Os06g30460.2 | 2-oxo acid dehydrogenases acyltransferase domain containing protein, expressed |  |
|  | LOC_Os04g19740.1 | transketolase, chloroplast precursor, putative, expressed |  |
|  | LOC_Os07g08840.1 | thioredoxin, putative, expressed | Q |
|  | LOC_Os07g37730.1 | NADH-ubiquinone oxidoreductase, mitochondrial precursor, putative, expressed |  |
|  | LOC_Os11g32500.1 | rubredoxin family protein, putative, expressed |  |
|  | LOC_Os01g27360.1 | glutathione S-transferase, putative, expressed |  |
|  | LOC_Os12g08260.3LOC_Os12g08260.4 | dehydrogenase E1 component domain containing protein, expressed |  |
|  | LOC_Os01g70990.1 | OsGrx_C6 - glutaredoxin subgroup III | Qex |
| 4  Peroxidase | LOC_Os06g33100.1 | peroxidase precursor, putative, expressed | Qex |
|  | LOC_Os01g73200.1 | peroxidase precursor, putative, expressed |  |
|  | LOC_Os07g48060.1 | peroxidase precursor, putative, expressed |  |
|  | LOC_Os03g22010.1 | peroxidase precursor, putative, expressed |  |
|  | LOC_Os07g44480.1 | peroxidase, putative, expressed |  |
|  | LOC_Os06g35480.1 | peroxidase precursor, putative, expressed | Qex |
|  | LOC_Os06g32990.1 | peroxidase precursor, putative, expressed | Qex |
|  | LOC_Os06g16350.1 | peroxidase precursor, putative, expressed |  |
|  | LOC_Os07g44460.1 | peroxidase precursor, putative, expressed |  |
|  | LOC_Os06g33080.1 | peroxidase precursor, putative, expressed | Qex |
| 5  Aspartic proteinase nepenthesin | LOC_Os09g38380.1 | aspartic proteinase nepenthesin, putative, expressed |  |
|  | LOC_Os10g39350.1 | aspartic proteinase nepenthesin, putative, expressed |  |
|  | LOC_Os03g08790.1 | aspartic proteinase nepenthesin precursor, putative, expressed |  |
|  | LOC_Os10g39260.1 | aspartic proteinase nepenthesin, putative, expressed |  |
|  | LOC_Os01g64840.1 | aspartic proteinase nepenthesin-1 precursor, putative, expressed | Q |
|  | LOC_Os10g39270.1 | nucleoid DNA-binding, putative, expressed |  |
|  | LOC_Os03g20290.1 | aspartic proteinase nepenthesin-1 precursor, putative, expressed |  |
|  | LOC_Os10g39390.1 | Eukaryotic aspartyl protease domain containing protein, expressed |  |
|  | LOC_Os10g39360.1 | aspartic proteinase nepenthesin precursor, putative, expressed |  |
| 6  Ubiquitin | LOC_Os05g36310.1 | ubiquitin conjugating enzyme protein, putative, expressed |  |
|  | LOC_Os01g60730.1LOC_Os01g60730.2 | ubiquitin conjugating enzyme protein, putative, expressed | Q |
|  | LOC_Os01g64620.1 | ubiquitin conjugating enzyme protein, putative, expressed | Q |
|  | LOC_Os02g48910.1 | ubiquitin conjugating enzyme protein, putative, expressed |  |
|  | LOC_Os02g38410.1 | ubiquitin conjugating enzyme protein, putative, expressed |  |
|  | LOC_Os09g31031.2 | ubiquitin conjugating enzyme protein, putative, expressed | Qex |
|  | LOC_Os09g27930.1 | ubiquitin conjugating enzyme protein, putative, expressed | Qex |
|  | LOC_Os01g13280.1 | ubiquitin conjugating enzyme protein, putative, expressed |  |
| 7  Starch | LOC_Os03g57980.1 | LTPL99 - Protease inhibitor/seed storage/LTP family protein precursor, expressed |  |
|  | LOC_Os04g33920.1 | LTPL102 - Protease inhibitor/seed storage/LTP family protein precursor, expressed | Q |
|  | LOC_Os10g41550.1 | beta-amylase, putative, expressed |  |
|  | LOC_Os01g63810.1 | starch binding domain containing protein, putative, expressed | Q |
|  | LOC_Os06g49970.2 | alpha-amylase precursor, putative, expressed |  |
| 8 Glucosyltransfera-se | LOC_Os06g16000.1 | UDP-glucoronosyl and UDP-glucosyl transferase, putative, expressed |  |
|  | LOC_Os07g10190.1 | glucosyltransferase, putative, expressed | Q |
|  | LOC_Os09g16030.1 | cytokinin-O-glucosyltransferase 1, putative, expressed |  |
|  | LOC_Os03g49550.1 | glucosyltransferase, putative, expressed |  |
|  | LOC_Os04g44250.1 | cytokinin-O-glucosyltransferase 3, putative, expressed |  |
| 9  Flavonol synthase | LOC_Os07g01340.1 | flavonol synthase/flavanone 3-hydroxylase, putative, expressed |  |
|  | LOC_Os08g30100.1 | flavonol synthase/flavanone 3-hydroxylase, putative, expressed |  |
|  | LOC_Os05g03640.1 | flavonol synthase/flavanone 3-hydroxylase, putative, expressed |  |
|  | LOC_Os03g58890.2 | flavonol synthase/flavanone 3-hydroxylase, putative, expressed |  |
|  | LOC_Os01g61610.1LOC_Os01g61610.2LOC_Os01g61610.3 | flavonol synthase/flavanone 3-hydroxylase, putative, expressed | Q |
| 10  Glycosyl hydrolases family | LOC_Os01g71820.1 | glycosyl hydrolases family 17, putative, expressed |  |
|  | LOC_Os05g31140.1 | glycosyl hydrolases family 17, putative, expressed |  |
|  | LOC_Os03g45390.1 | glucan endo-1,3-beta-glucosidase precursor, putative, expressed |  |
|  | LOC_Os01g71810.1 | glycosyl hydrolases family 17, putative, expressed |  |
| 11  MYB family transcription factor | LOC_Os02g46030.1 | MYB family transcription factor, putative, expressed |  |
|  | LOC_Os06g51260.1 | MYB family transcription factor, putative, expressed |  |
|  | LOC_Os04g42950.1 | MYB family transcription factor, putative, expressed |  |
|  | LOC_Os06g01670.1 | myb-like DNA-binding domain containing protein, expressed |  |
| 12  Gibberellin receptor GID1L2 | LOC_Os11g13670.1 | gibberellin receptor GID1L2, putative, expressed |  |
|  | LOC_Os07g06840.1 | gibberellin receptor GID1L2, putative, expressed | Q |
|  | LOC_Os07g06860.1 | gibberellin receptor GID1L2, putative, expressed | Q |
|  | LOC_Os06g20200.1 | gibberellin receptor GID1L2, putative, expressed |  |
| 13  O-methyltransferase | LOC_Os11g20090.1 | O-methyltransferase, putative, expressed |  |
|  | LOC_Os11g12760.1 | O-methyltransferase, putative, expressed |  |
|  | LOC_Os05g43940.1 | O-methyltransferase, putative, expressed |  |
|  | LOC_Os12g25490.1 | O-methyltransferase, putative, expressed |  |
| 14 Phosphatidylethanolamine-binding | LOC_Os04g33570.1 | RCN4 Centroradialis-like1 homologous to TFL1 gene; contains Pfam profile PF01161: Phosphatidylethanolamine-binding protein, expressed | Q |
|  | LOC_Os04g41130.1 | osFTL6 FT-Like6 homologous to Flowering Locus T gene; contains Pfam profile PF01161: Phosphatidylethanolamine-binding protein, expressed | Q |
|  | LOC_Os10g01110.1 | OsSCP44 - Putative Serine Carboxypeptidase homologue, expressed |  |
|  | LOC_Os11g18870.1 | osFTL11 FT-Like11 homologous to Flowering Locus T gene; contains Pfam profile PF01161: Phosphatidylethanolamine-binding protein, expressed |  |
| 15  Aldo/keto reductase family | LOC_Os04g37490.1 | oxidoreductase, aldo/keto reductase family protein, putative, expressed | Q |
|  | LOC_Os03g13390.2 | oxidoreductase, aldo/keto reductase family protein, putative, expressed |  |
| 16  Cytokinin dehydrogenase | LOC_Os06g35650.1 | reticuline oxidase-like protein precursor, putative, expressed | Qex |
|  | LOC_Os01g56810.1 | cytokinin dehydrogenase precursor, putative, expressed | Qex |
| 17  Cysteine synthase/indole-3-glycerol phosphate lyase | LOC_Os06g36850.1 | cysteine synthase, putative, expressed | Q |
|  | LOC_Os03g58300.1 | indole-3-glycerol phosphate lyase, chloroplast precursor, putative, expressed |  |

Each member of the modules is annotated in the column based on MSU7. When the gene is mapped to the QTL region, the QTL column is marked as Q. When the gene is mapped to the QTL extended region, the QTL column is marked as Qex.

Table B. Co-expression analysis of Modules 2-17 in salt-tolerance network.

| Module | Average inside-module Pearson correlation coefficient | Average Pearson correlation coefficient between inside and outside the module |
| --- | --- | --- |
| 2 | 0.4920 | 0.2565 |
| 3 | 0.4810 | 0.2595 |
| 4 | 0.6592 | 0.2417 |
| 5 | 0.3237 | 0.2387 |
| 6 | 0.5252 | 0.2776 |
| 7 | 0.4082 | 0.2142 |
| 8 | 0.4176 | 0.2514 |
| 9 | 0.4540 | 0.2093 |
| 10 | 0.6221 | 0.3100 |
| 11 | 0.6534 | 0.3049 |
| 12 | 0.5580 | 0.3471 |
| 13 | 0.7597 | 0.3152 |
| 14 | 0.5972 | 0.3845 |
| 15 | 0.7440 | 0.3455 |
| 16 | 0.8295 | 0.3518 |
| 17 | 0.8913 | 0.3558 |

Average Pearson correlation coefficients of expression of the genes inside module were calculated, and then average Pearson correlation coefficients of expression of gene between genes inside a module and randomly selected genes outside the module were calculated.

Table C. GO term enrichment analysis on genes in Module 2 by AgriGO.

| GO term | Ontology | Description | Number in selected gene set | Number in Background | p-value | FDR |
| --- | --- | --- | --- | --- | --- | --- |
| GO:0006118 | P | electron transport | [16](http://bioinfo.cau.edu.cn/agriGO/termDetail.php?session=180528139&GO=GO:0006118) | 346 | 4.9e-26 | 4.9e-26 |
| GO:0004497 | F | monooxygenase activity | [16](http://bioinfo.cau.edu.cn/agriGO/termDetail.php?session=180528139&GO=GO:0004497) | 196 | 4.6e-30 | 1.5e-28 |
| GO:0020037 | F | heme binding | [16](http://bioinfo.cau.edu.cn/agriGO/termDetail.php?session=180528139&GO=GO:0020037) | 205 | 9.7e-30 | 1.6e-28 |
| GO:0046906 | F | tetrapyrrole binding | [16](http://bioinfo.cau.edu.cn/agriGO/termDetail.php?session=180528139&GO=GO:0046906) | 217 | 2.5e-29 | 2.7e-28 |
| GO:0005506 | F | iron ion binding | [16](http://bioinfo.cau.edu.cn/agriGO/termDetail.php?session=180528139&GO=GO:0005506) | 432 | 1.7e-24 | 1.4e-23 |
| GO:0016491 | F | oxidoreductase activity | [16](http://bioinfo.cau.edu.cn/agriGO/termDetail.php?session=180528139&GO=GO:0016491) | 1141 | 7.1e-18 | 4.7e-17 |
| GO:0046914 | F | transition metal ion binding | [16](http://bioinfo.cau.edu.cn/agriGO/termDetail.php?session=180528139&GO=GO:0046914) | 1814 | 7.9e-15 | 4.4e-14 |
| GO:0046872 | F | metal ion binding | [16](http://bioinfo.cau.edu.cn/agriGO/termDetail.php?session=180528139&GO=GO:0046872) | 2480 | 7.7e-13 | 3.7e-12 |
| GO:0043169 | F | cation binding | [16](http://bioinfo.cau.edu.cn/agriGO/termDetail.php?session=180528139&GO=GO:0043169) | 2582 | 1.4e-12 | 5.1e-12 |
| GO:0043167 | F | ion binding | [16](http://bioinfo.cau.edu.cn/agriGO/termDetail.php?session=180528139&GO=GO:0043167) | 2584 | 1.4e-12 | 5.1e-12 |
| GO:0003824 | F | catalytic activity | [16](http://bioinfo.cau.edu.cn/agriGO/termDetail.php?session=180528139&GO=GO:0003824) | 8329 | 5.8e-06 | 1.9e-05 |
| GO:0005488 | F | binding | [16](http://bioinfo.cau.edu.cn/agriGO/termDetail.php?session=180528139&GO=GO:0005488) | 8681 | 9.3e-06 | 2.8e-05 |

Ontology “P” indicates Biological Process, Ontology “F” indicates Molecular Function, and Ontology “C” indicates Cellular Component. “Number in selected gene set” is the number of genes in the query gene list. “Number in background” is the number of genes in the proteome. P-value represents the statistical significance of the gene enrichment test. FDR means False Discovery Rate.

Table D. GO term enrichment analysis on gene of Module4 by AgriGO.

| GO term | Ontology | Description | Number in selected gene set | Number in Background | p-value | FDR |
| --- | --- | --- | --- | --- | --- | --- |
| GO:0006979 | P | response to oxidative stress | 10 | 156 | 2.40E-05 | 0.011 |
| GO:0006118 | P | electron transport | [10](http://bioinfo.cau.edu.cn/agriGO/termDetail.php?session=879355884&GO=GO:0006118) | 346 | 1e-16 | 5.7e-16 |
| GO:0042221 | P | response to chemical stimulus | [10](http://bioinfo.cau.edu.cn/agriGO/termDetail.php?session=879355884&GO=GO:0042221) | 648 | 5e-14 | 1.9e-13 |
| GO:0006950 | P | response to stress | [10](http://bioinfo.cau.edu.cn/agriGO/termDetail.php?session=879355884&GO=GO:0006950) | 885 | 1e-12 | 3e-12 |
| GO:0050896 | P | response to stimulus | [10](http://bioinfo.cau.edu.cn/agriGO/termDetail.php?session=879355884&GO=GO:0050896) | 1462 | 1.3e-10 | 2.9e-10 |
| GO:0004601 | F | peroxidase activity | [10](http://bioinfo.cau.edu.cn/agriGO/termDetail.php?session=879355884&GO=GO:0004601) | 107 | 6.5e-22 | 1.3e-20 |
| GO:0016684 | F | oxidoreductase activity, acting on peroxide as acceptor | [10](http://bioinfo.cau.edu.cn/agriGO/termDetail.php?session=879355884&GO=GO:0016684) | 107 | 6.5e-22 | 1.3e-20 |
| GO:0016209 | F | antioxidant activity | [10](http://bioinfo.cau.edu.cn/agriGO/termDetail.php?session=879355884&GO=GO:0016209) | 120 | 2.1e-21 | 3e-20 |
| GO:0020037 | F | heme binding | [10](http://bioinfo.cau.edu.cn/agriGO/termDetail.php?session=879355884&GO=GO:0020037) | 205 | 5.1e-19 | 5.3e-18 |
| GO:0046906 | F | tetrapyrrole binding | [10](http://bioinfo.cau.edu.cn/agriGO/termDetail.php?session=879355884&GO=GO:0046906) | 217 | 9.2e-19 | 7.6e-18 |
| GO:0005506 | F | iron ion binding | [10](http://bioinfo.cau.edu.cn/agriGO/termDetail.php?session=879355884&GO=GO:0005506) | 432 | 9.1e-16 | 6.3e-15 |
| GO:0016491 | F | oxidoreductase activity | [10](http://bioinfo.cau.edu.cn/agriGO/termDetail.php?session=879355884&GO=GO:0016491) | 1141 | 1.2e-11 | 7e-11 |
| GO:0046914 | F | transition metal ion binding | [10](http://bioinfo.cau.edu.cn/agriGO/termDetail.php?session=879355884&GO=GO:0046914) | 1814 | 9.5e-10 | 4.9e-09 |
| GO:0046872 | F | metal ion binding | [10](http://bioinfo.cau.edu.cn/agriGO/termDetail.php?session=879355884&GO=GO:0046872) | 2480 | 1.7e-08 | 7.7e-08 |
| GO:0043169 | F | cation binding | [10](http://bioinfo.cau.edu.cn/agriGO/termDetail.php?session=879355884&GO=GO:0043169) | 2582 | 2.4e-08 | 9.1e-08 |
| GO:0043167 | F | ion binding | [10](http://bioinfo.cau.edu.cn/agriGO/termDetail.php?session=879355884&GO=GO:0043167) | 2584 | 2.4e-08 | 9.1e-08 |
| GO:0003824 | F | catalytic activity | [10](http://bioinfo.cau.edu.cn/agriGO/termDetail.php?session=879355884&GO=GO:0003824) | 8329 | 0.00034 | 0.0012 |
| GO:0005488 | F | binding | [10](http://bioinfo.cau.edu.cn/agriGO/termDetail.php?session=879355884&GO=GO:0005488) | 8681 | 0.00046 | 0.0015 |

Ontology “P” indicates Biological Process, Ontology “F” indicates Molecular Function, and Ontology “C” indicates Cellular Component. “Number in selected gene set” is the number of genes in the query gene list. “Number in background” is the number of genes in the proteome. P-value represents the statistical significance of the gene enrichment test. FDR means False Discovery Rate.

| 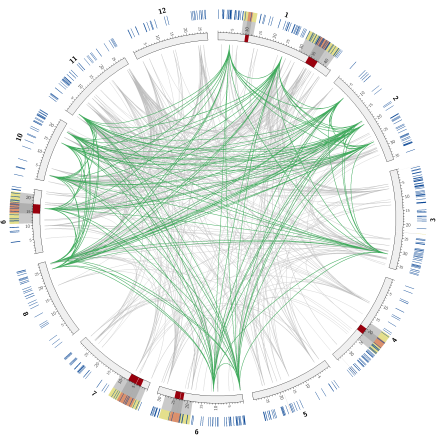 | 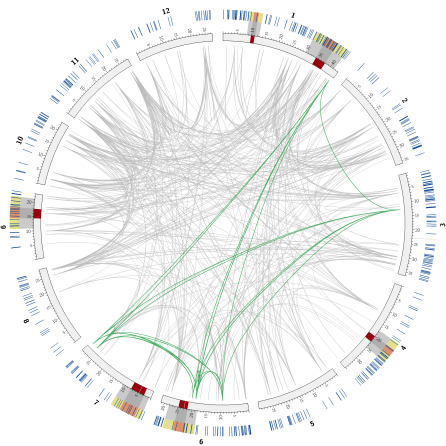 | 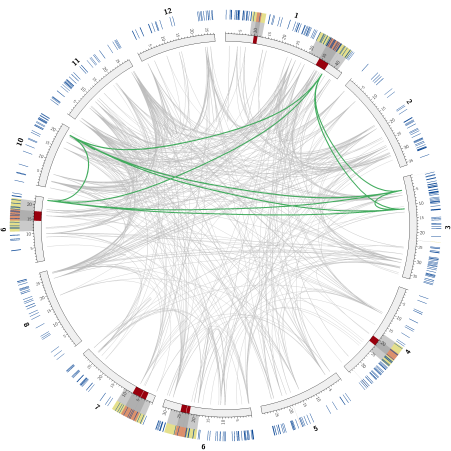 |
| --- | --- | --- |
| Figure. A(a) | Figure. A(b) | Figure. A(c) |
| 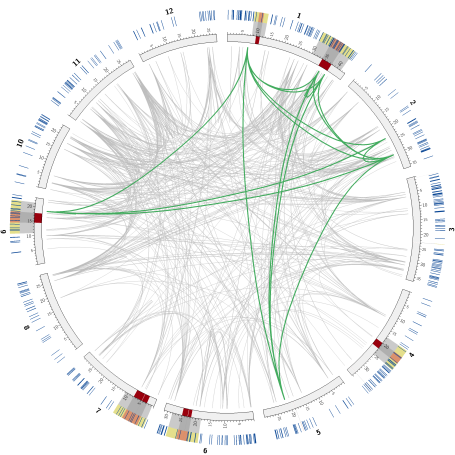 | 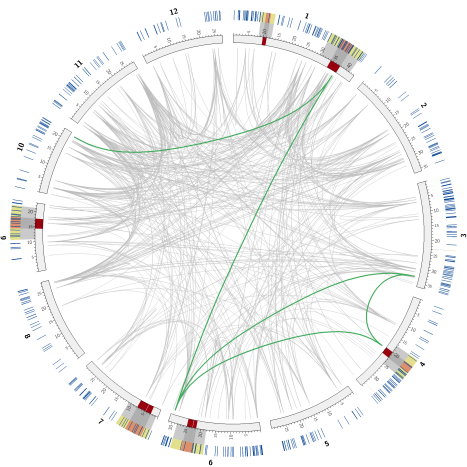 | 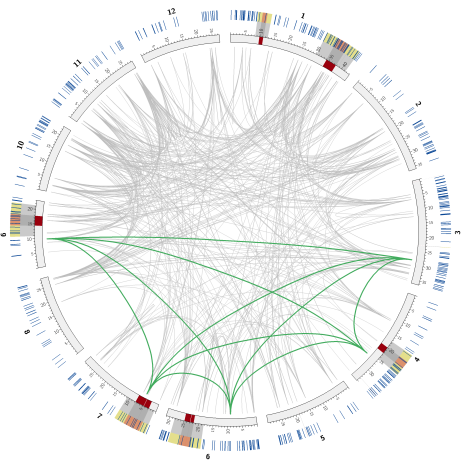 |
| Figure. A(d) | Figure. A(e) | Figure. A(f) |
| 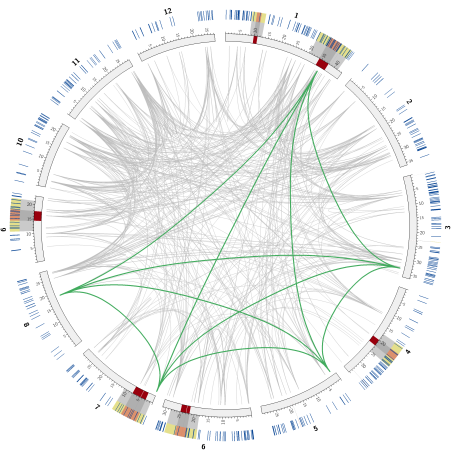 | 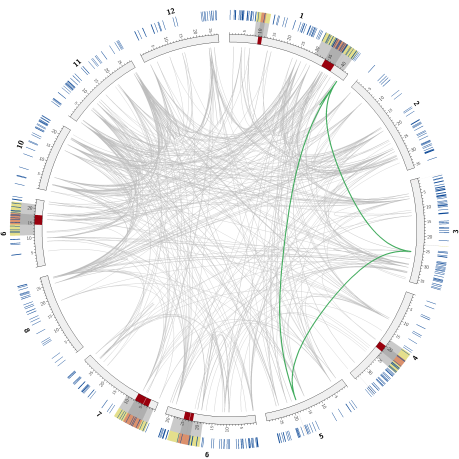 | 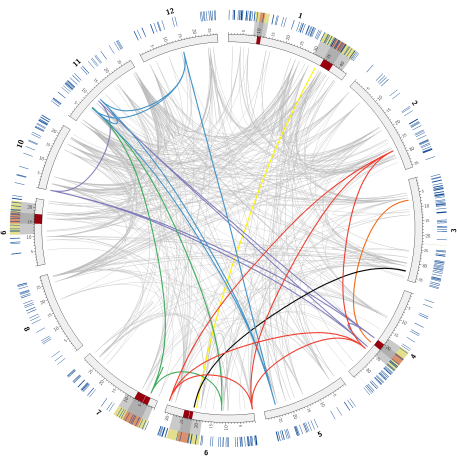 |
| Figure. A(g) | Figure. A(h) | Figure. A(i) |

Figure A(a)-(i): interactions among genes in a module on rice genome. (a) Module 2; (b) Module 4; (c) Module 5; (d) Module 6; (e) Module 7; (f) Module 8; (g) Module 9; (h) Module 10. (i) Module 11-17. Red lines show interactions of Module 11, green lines show interactions of Module 12, blue lines show interactions of Module 13, purple lines show interactions of Module 14, orange lines show interactions of Module 15, yellow lines show interactions of Module 16, and black lines show interactions of Module 17.

**References:**

1. S Ouyang, W Zhu, J Hamilton, H Lin, M Campbell et al. (2007) The TIGR Rice Genome Annotation Resource: improvements and new features. Nucleic Acids Research 35:D883-D887.

2. F Guengerich (2008). Cytochrome p450 and chemical toxicology. Chem. Res. Toxicol. 21(1): 70–83.

3. R Sigel, A Sigel, H Sigel (2007). The Ubiquitous Roles of Cytochrome P450 Proteins: Metal Ions in Life Sciences. New York: Wiley. ISBN 0-470-01672-8.

4. N Kitahata, S Saito, Y Miyazawa, T Umezawa, Y Shimada et al. (2005) Chemical regulation of abscisic acid catabolism in plants by cytochrome P450 inhibitors, Bioorganic & Medicinal Chemistry, 13(14):4491-4498.

5. M Seki, M Narusaka, J Ishida, T Nanjo, M Fujita (2002) Monitoring the expression profiles of 7000 Arabidopsis genes under drought, cold and high-salinity stresses using a full-length cDNA microarray, The Plant Journal, 31(3):279-292.

6. Y Narusaka, M Narusaka, M Seki, T Umezawa, J Ishida et al. (2004) Crosstalk in the responses to abiotic and biotic stresses in Arabidopsis: Analysis of gene expression in cytochrome P450 gene superfamily by cDNA microarray, Plant Molecular Biology, 55(3):327-342.

7. M Dionisio-Sese, S Tobita (1998) Antioxidant responses of rice seedlings to salinity stress, Plant Science, 135(1):1-9.

8. A Kalir, G Omri, A Poljakoff-Mayber (1984) Peroxidase and catalase activity in leaves of Halimione portulacoides exposed to salinityPhysiologia Plantarum, 62(2):238-244.

9. J Negrel, J Lherminier (1987) Peroxidase-mediated integration of tyramine into xylem cell walls of tobacco leaves, Planta, 172:494-501.

10. A Polle, T Otter, F Seifert (1994) Apoplastic peroxidases and lignification in needles of Norway spruce (Picea abies L.), Plant Physiol., 106:53-60.

11. K Espelie, V Franceschi, P Kolattukudy (1986) Immunocytochemical localization and time course of appearance of an anionic peroxidase associated with suberization in wound-healing potato tuber tissue, Plant Physiol., 81:487-492.

12. M Guevara, P Veríssimo, E Pires, C Faro, GR Daleo (2004) Potato aspartic proteases: induction, antimicrobial activity and substrate specificity, Journal of Plant Pathology, 86(3):233-238.

13. M Guevara, C Oliva, M Huarte, G Daleo (2002) An Aspartic Protease With Antimicrobial Activity is Induced after Infection and Wounding in Intercellular Fluids of Potato Tubers, European Journal of Plant Pathology, 108(2):131-137.

14. B Bartel,V Citovsky (2012) Focus on Ubiquitin in Plant Biology, Plant Physiology, 160:1.

15. H Bae, S Kim, S Cho, B Kang, W Kim (2011) Overexpression of OsRDCP1, a rice RING domain-containing E3 ubiquitin ligase, increased tolerance to drought stress in rice (Oryza sativa L.), Plant Science, 180:6:775-782.

16. D Fiol, E Sanmarti, A Lim, D Kultz (2011) A novel GRAIL E3 ubiquitin ligase promotes environmental salinity tolerance in euryhaline tilapia, Biochimica et Biophysica Acta (BBA) - General Subjects, 1810(4):439-445.

17. J Ko, S Yang, K Han (2006) Upregulation of an Arabidopsis RING-H2 gene, XERICO, confers drought tolerance through increased abscisic acid biosynthesis. Plant Journal, 47:343-355.

18. K Dreher, J Callis(2007) Ubiquitin hormones and biotic stress in plants, Ann. Bot. (Lond.), 99:787-822.

19. R Dubey, A Singh (1999) Salinity Induces Accumulation of Soluble Sugars and Alters the Activity of Sugar Metabolising Enzymes in Rice Plants, Biologia plantarum, 42(2):233-239.

20. P Wattana, T Maysaya (2008) Effect of salinity stress on growth and carbohydrate metabolism in three rice (Oryza sativa L.) cultivars differing in salinity tolerance, Indian journal of experimental Biology, IJEB 46:736-742.

21. A Krapp, M Stitt (1995) An evaluation of direct and indirect mechansims for the 'sink-regulation' of photosynthesis in spinach: changes in gas exchange, carbohydrates, metabolites, enzyme activities and steady-state transcript levels after cold-gridling source leaves, Planta, 195(3):313-323.

22. M Balibera, J Amico, M Bolarin, F Perez-Alfocea (2000) Carbon partitioning and sucrose mettabolism in tomato plants growing udner salinity, Phsiol Plant, 110(4):503-511.

23. Y Qi, N Kawano, Y Yamauchi, J Ling, D Li (2005) Identification and cloning of a submergence-induced gene OsGGT (glycogenin glucosyltransferase) from rice (Oryza sativa L.) by suppression subtractive hybridization. Planta, 221(3):437-445.

24. M Uddin, M Kihara, L Yin, M Perveen, K Tanaka (2012), Expression and subcellular localization of antiporter regulating protein OsARP in rice induced by submergence, salt and drought stresses, African Journal of Biotechnology 11(65):12849-12855.

25. A Fini, C Brunetti, M Ferdinando, F Ferrini, M Tattini (2011), Stress-induced flavonoid biosynthesis and the antioxidant machinery of plants, Plant Signal Behav, 6(5):709-711.

26. G Agatia, S Biricoltib, L Guidic, F Ferrinib, A Finib et al. (2011) The biosynthesis of flavonoids is enhanced similarly by UV radiation and root zone salinity in L. vulgare leaves,Journal of Plant Physiology, 168(3):204-212.

27. T Tsuda, K Shiga, K Ohshima, S Kuwakishi, T Osuwu (1996) Inhibition of lipid peroxidation and the active oxygen radical scavenging effect of anthocyanin pigments isolated from Phaseoks vulgaris L. Biochemi. Pharma., 52, 1033-1039.

28. J Zhou, X Wang, Y Jiao, Y Qin, X Liu et al. (2007) Global genome expression analysis of rice in response to drought and high-salinity stresses in shoot, flag leaf, and panicle,Plant Mol Biol,63:591-608.

29. H Magome, S Yamaguchi, A Hanada, Y Kamiya, K Oda(2008) The DDF1 transcriptional activator upregulates expression of a gibberellin-deactivating gene, GA2ox7, under high-salinity stress in Arabidopsis,The Plant Journal, 56(4):613-626.

30. I Ogawa, H Nakanishi, S Mori, N Nishizawa (2009)Time course analysis of gene regulation under cadmium stress in rice, Plant and soil, 325:(1-2):97-108.

31. H Mizuno, H Kawahigashi, Y Kawahara, H Kanamori, J Ogata et al.(2012)Global transcriptome analysis reveals distinct expression among duplicated genes during sorghum-Bipolarissorghicola interaction, BMC Plant Biology, 12:121.

32. Pollard M, Beisson F, Li Y, Ohlrogge JB (2008) Building lipid barriers: biosynthesis of cutin and suberin. Trends Plant Sci, 13(5):236–246.

33. E Sheveleva, W Chmara, H Bohnert, R Jensen (1997) Increased salt and drought tolerance by d-ononitol production in transgenic Nicotiana tabacum L. Plant Physiol 115:1211–1219.

34. H Walia, C Wilson, A Wahid, P Condamine, X Cui et al. (2006) Expression analysis of barley (Hordeum vulgare L.) during salinity stress, Functional & Integrative Genomics, 6(2):143-156.

35. R Caesar, A Blomberg (2004) The stress-induced Tfs1p requires NatB-mediated acetylation to inhibit carboxypeptidase Y and to regulate the protein kinase A pathway, Journal of Biological Chemistry,279(37):38532-38543.

36. Z Turóczy, P Kis, K Török, M Cserháti, Á Lendvai et al.(2011) Overproduction of a rice aldo–keto reductase increases oxidative and heat stress tolerance by malondialdehyde and methylglyoxal detoxification, Plant Molecular Biology, 75(4-5):399-412.

37. P Simpson, C Tantitadapitak, AM Reed, O Mather, C Bunce et al.(2009) Characterization of Two Novel Aldo–KetoReductases from Arabidopsis: Expression Patterns, Broad Substrate Specificity, and an Open Active-Site Structure Suggest a Role in Toxicant Metabolism Following Stress, Journal of Molecular Biology, 392(2):465–480.

38. L Vysotskaya, A Korobova, S Veselov, I Dodd, G Kudoyarova (2009) ABA mediation of shoot cytokinin oxidase activity: assessing its impacts on cytokinin status and biomass allocation of nutrient-deprived durum wheat, Functional Plant Biology 36(1):66-72.

39. R Wang, S Pandey, S Li, T Gookin, Z Zhao et al. (2011) Common and unique elements of the ABA-regulated transcriptome of Arabidopsis guard cells, BMC Genomics, 12:216.

40. N Sreenivasulu, V Harshavardhan, G Govind, C Seiler, A Kohli(2012), Contrapuntal role of ABA: Does it mediate stress tolerance or plant growth retardation under long-term drought stress?, Gene,506(2):265-273.

41. R Jacoby, A Millar, N Taylor (2010) Wheat mitochondrial proteomes provide new links between antioxidant defense and plant salinity tolerance, Journal of Proteome Research, 9(12):6595-604.

42. Q Yang, Y Wang, J Zhang, W Shi, C Qian, X Peng (2007) Identification of aluminum©\responsive proteins in rice roots by a proteomic approach: Cysteine synthase as a key player in Al response, Proteomics, 7(5):737-749.

43. H Mizuno, Y Kawahara, H Sakai, H Kanamori, H Wakimoto et al. (2010) Massive parallel sequencing of mRNA in identification of unannotated salinity stress-inducible transcripts in rice (Oryza sativa L.),BMC Genomics, 11:683.

44. H Less, G Galili (2008) Principal transcriptional programs regulating plant amino acid metabolism in response to abiotic stresses. Plant Physiol, 147(1):316-330.
