# Supplementary material for: A Computational Systems Biology Study for Understanding Salt Tolerance Mechanism in Rice
Source: PLoS One. 2013 Jun 7;8(6):e64929. doi: 10.1371/journal.pone.0064929 (PMC3676415; doi:10.1371/journal.pone.0064929)

**Document S5**

**Choice of gene list using improved volcano plot**

We proposed an improved volcano plot method to choose genes in this dataset. In the improved volcano plot, the t-test p-value, instead of the original volcano plot, was used as Y-axis, and a merged value *MergeValue* obtained information from SVM-RFE method, instead of fold-change, which was used as X-axis.

The SVM-RFE method was introduced for ranking genes from gene expression data for cancer classification [1]. In the point of view from machine learning, feature means probe site that was designed to represent genes in microarray. In the SVM-RFE algorithm, features are individually eliminated based on their least significance for classification in SVM training. The objective function for classification *J* is defined as:

$J=\left\|w\right\|^{2}/2$ (1)

in which $w$is the kernel width computed by SVM. The Optimal Brain Damage (OBD) algorithm [2] is used to approximate the change of *J* when removing *i*-th feature by expanding *J* in the Taylor series to the second order. At the optimum of *J*, the first term could be neglected, yielding:

$\Delta J(i)=(1/2)\frac{\partial^{2}J}{\partial w_{i}^{2}}(\Delta w_{i})^{2}$ (2)

The change in weight$\Delta w_{i}=w_{i}$corresponds to removing the *i*-th feature. $\Delta J(i)$ could be used as the weight pruning criterion, and linear discriminate functions whose cost function *J* is a quadratic function of $w_{i}$, so these two criteria are equivalent. Usually we use$w_{i}^{2}$as the ranking criterion for evaluating the impact on classification [3]. In this process, we removed the feature with the smallest$w_{i}^{2}$iteratively in a backward elimination manner and obtain a ranked feature list at last.

Many methods of feature/variable selection are susceptible to small permutation of the experiment conditions [1]. The widely used general bootstrap method to get robust subset for overcoming this shortcoming fails in this type of gene expression microarray dataset because there are so few samples for training. In this case, a revised bootstrap procedure using valuable training samples was introduced to sort genes and evaluate the classification results.

In the bootstrap procedure, for *M* samples in a dataset, each time we selected *m* samples to construct the training set (*m*<=*M*), and then performed an SVM-RFE procedure to get one ranked gene list. Then we repeated the procedure for *n* times, each time selecting different *m* samples as a training set, where we obtained a set of *n* rank gene lists.

A score function *RankScore* is defined to incorporate the information of gene ranks variously in each gene list to evaluate the total ranked gene list of all the genes. The *RankScore_ij_* is defined for gene *i* in rank list *j* as

$RankScore_{ij}=\frac{d+1-p_{ij}}{d},1\leq p_{ij}\leq d$ (3)

where *d* represents the number of all features, and *p_ij_* is the ranked position of gene *i* in rank list *j*. The *RankScore_i_* of each gene *i* is defined to summarize all the scores of *n* rank lists:

$RankScore_{i}=\sum_{j=1}^{n} \frac{d+1-p_{ij}}{d},1\leq p_{ij}\leq d$ (4)

In the definition of *RankScore*, each rank list was used to deal with equal weights for the equal partition of dataset in the bootstraps; each gene which is placed on top of the lists could get high values, though the weight is scaled by the number of features *d*, that implicitly enlarged the weight of position ranked on the top and reduced the weight of position ranked behind. After obtaining all the genes with high *RankScore*, we get the final gene list *S_final_*, which represents the importance of genes just by sorting.

The algorithm of the bootstraps procedure is described as follows:

Step 1: Define ranked feature list *S*={}, where index *i*=1, and *M* is the number of total samples.

Step 2: Construct data set *T_i_* with *m* samples randomly selected from total M samples.

Step 3: Perform a standard SVM-RFE procedure on data set *T_i_*

Step 4: Obtain rank list *S_i_*

Step 5: If *i* does not equal to *n,* then set *i=i+*1, and go back to Step 2 or else go to Step 6.

Step 6: Calculate *RankScore* for all the genes in all rank lists, *S*, then sort the *RankScore* in descending order and get the final gene list *S_final_*.

In the Bootstraps procedure, each time we selected 15 samples randomly from the balanced 9 positive and 9 negative samples in the dataset to perform the SVM-RFE procedure. Repeat this Bootstraps procedure 100 times and obtain 100 gene lists.

To evaluate the performance of SVM-RFE, we constructed an SVM classifier in a leave-one-out procedure. The accuracy of an individual classifier ranked by features/genes is shown in Figure S6(a). We then used a sliding-window with a width of 100 sorted features to obtain the average trend in Figure S6(b). This average trend was fitted as follows:

$f(x)=e^{-0.0001371\times(x+17240)}+4.005$ (5)

where *x* presents the rank of the feature used in the classifier and$f(x)$represents the accuracy obtained by the classifier. The fitting curve is a monotone decreasing function. Half of the maximum accuracy was achieved at the 5054-ranked probe set. Hence, we chose 5054 features as a candidate threshold to evaluate the performance.

We define *ConsistencyValue* as the function of *x* in Equation (6) to gather and format rank information of ranked gene lists obtained by SVM-RFE:

$ConsistencyValue=\log(x+1)/\log101$ (6)

where *x* equals the sum of times a specific gene occurs in top 5054 of 100 obtained gene lists from bootstrap SVM-RFE, $x\in[0,100]$and its related$ConsistencyValue\in[0,1]$, which reveals the consistency of genes appearing in the gene lists and is another important measurement of ranked gene lists obtained from SVM-RFE. Using the fitted curve defined by Equation (5), we defined *RankScore* in Equation (7).

$RankValue=(\exp(-a\times(x+b))+c-lowbond)/height$ (7)

which depended heavily on the fitted curve. In this paper, *a* equals 0.0001371, *b* equals 1.724e+004, *c* equals 4.005, *lowbond* equals 4.005, which is the smallest value the curve achieved, height equals 0.0941, which is the height of the curve. *x* equals the rank position sorted by Bootstraps SVM-RFE *RankScore*,$x\in[1,57258]$ and its related $RankValue\in[0,1]$. *RankValue* collects the ranked information obtained from SVM-RFE and fitted curve of accuracy. In order to demonstrate the balanced performance of the ranking results, we defined *MergeValue,* which is *ConsistencyValue* multipled by *RankValue* as follows:

$MergeValue=ConsistencyValue\times RankValue$ (8)

where *MergeValue* depicts the rank attributes in machine learning overall. A larger value of gene means its higher importance in the specific trait, i.e., salt tolerance here. Other than the fold change dimension in volcano plot, the improved volcano plot kept the statistical p-value dimension and used *MergeValue* to replace fold change.

# References

1. Guoyon I, Elisseeff A (2003) An introduction to variable and feature selection. Journal of Machine Learning Research 3:1157-1182.
2. LeCun Y, Denker J, Solla S, Howard R, Jackel L (1990) Optimal brain damage, Advances in Neural Infromation Processing Systems, D.S. Touretzky, Ed. Mateo, CA: Morgan Kaufmann.
3. Guyon I, Weston J, Barnhill S, Vapnik V (2002) Gene selection for cancer classification using support vector machines, Machine Learning 46:389-422.

**Figure S6. The performance of single feature ranked by SVM-RFE.** (a) Accuracy of individual SVM classifier. (b) Average accuracy among SVMs with a slide window of 100 features. In both graphs, the horizontal axis represents the ranking of the feature/gene by *RankScore* obtained by bootstraps SVM-RFE. The vertical axis depicts SVM classifier accuracy in percentage in the Leave-One-Out procedure.


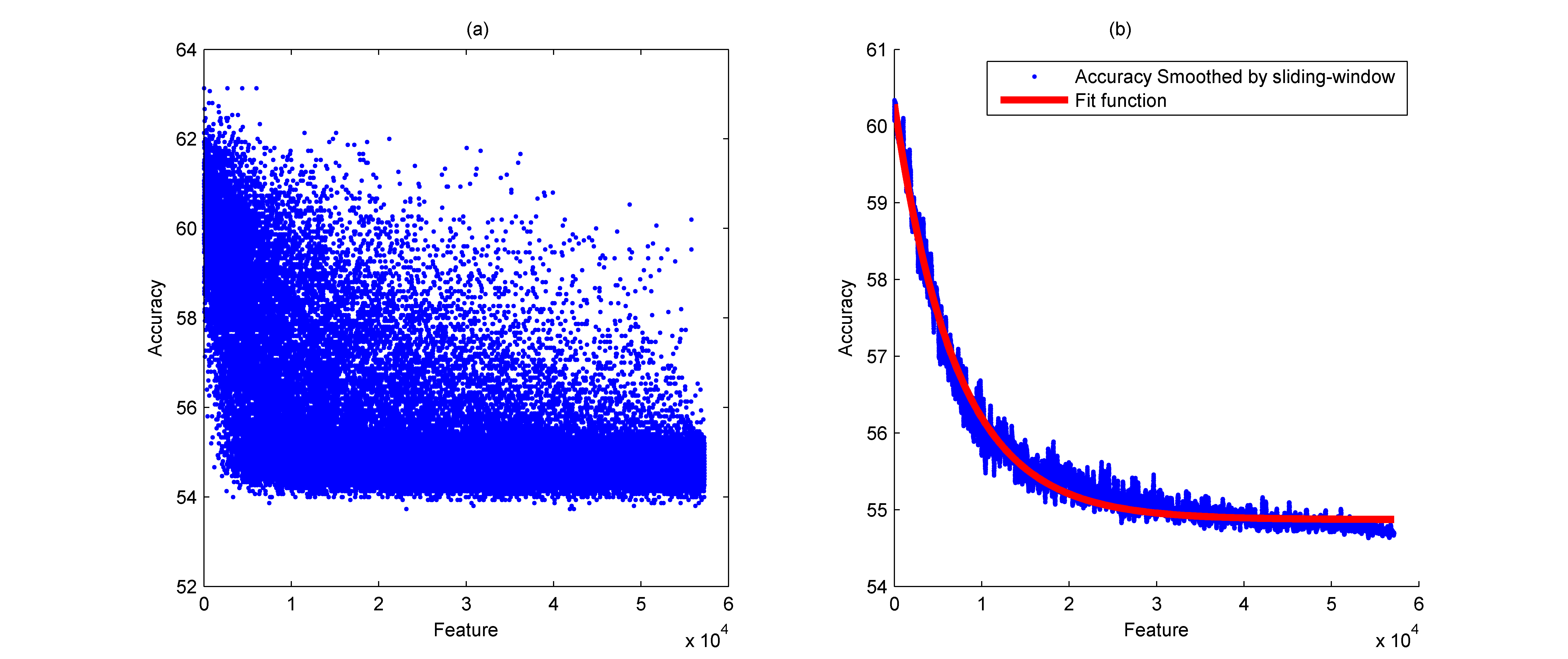

Supplement: Document S5 — Choice of gene list using improved volcano plot. (DOC) [file pone.0064929.s009.doc]
